# Red and far-red cleavable fluorescent dyes for self-labelling enzyme protein tagging and interrogation of GPCR co-internalization†

**DOI:** 10.1039/d4cb00209a

**Published:** 2024-11-18

**Authors:** Kilian Roßmann, Ramona Birke, Joshua Levitz, Ben Jones, Johannes Broichhagen

**Affiliations:** a Leibniz-Forschungsinstitut für Molekulare Pharmakologie (FMP) Berlin 13125 Germany broichhagen@fmp-berlin.de; b Department of Biochemistry, Weill Cornell Medicine New York NY USA; c Section of Endocrinology and Investigative Medicine, Department of Metabolism, Digestion and Reproduction, Imperial College London London W12 0NN UK

## Abstract

Post-labelling cleavable substrates for self-labelling protein tags, such as SNAP- and Halo-tags, can be used to study cell surface receptor trafficking events by stripping dyes from non-internalized protein pools. Since the complexity of receptor biology requires the use of multiple and orthogonal approaches to simultaneously probe multiple receptor pools, we report the development of four membrane impermeable probes that covalently bind to either the SNAP- or the Halo-tag in the red to far-red range. These molecules bear a disulfide bond to release the non-internalized probe using the reducing agent sodium 2-mercaptoethane sulfonate (MESNA). As such, our approach allows the simultaneous visualization of multiple internalized cell surface proteins in two colors which we showcase using G protein-coupled receptors. We use this approach to detect internalized group II metabotropic glutamate receptor (mGluRs), homo- and heterodimers, and to reveal unidirectional crosstalk between co-expressed glucagon-like peptide 1 (GLP1R) and glucose-dependent insulinotropic polypeptide receptors (GIPR). In these applications, we translate our method to both high resolution imaging and quantitative, high throughput assays, demonstrating the value of our approach for a wide range of applications.

## Abstract 2

SNAP- and Halo-tagged cell surface receptors are labelled, internalized and stripped from the extracellular pool using the reductant MESNA, allowing for two-color receptor co-internalization measurements and quantification.

## Graphical abstract

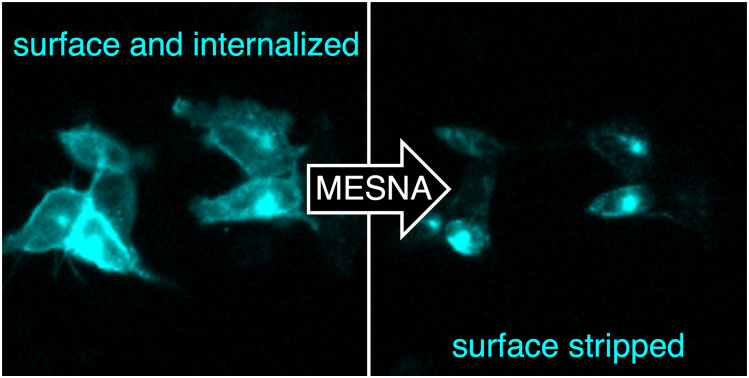

## Introduction

The dynamic equilibrium between the cell surface and intracellular compartments is central to the biological function of membrane proteins. Understanding the intricate processes of endocytosis and trafficking of membrane proteins in live cells is crucial for unraveling the mechanisms underlying cellular signaling dynamics^1^ which control biological functions including nutrient uptake,^2,3^ signaling,^4,5^ neuromodulation,^6^ immune response,^7^ and the maintenance of cell homeostasis.^8^ Importantly, membrane protein trafficking dysregulation has been implicated in cancer, neurodegenerative disease, and immune system disorders.^8–12^ Therefore, studying these phenomena in depth may allow us to glean valuable insights to drive the development of therapeutic strategies to modulate receptor signaling and restore normal cellular function.

The ability to study the internalization and trafficking of cell surface proteins has been greatly enhanced by the development and application of self-labelling protein tags, such as the SNAP- and Halo-tag.^13–16^ A bio-orthogonal handle, such as *O*^6^-benzylguanine (BG) or a chloroalkane (CA), can specifically label the SNAP- or Halo-tag, respectively, and when fused to a fluorophore, enable the visualization and tracking of proteins by microscopy. While many strategies exist to specifically label surface *versus* intracellular pools,^17^ it remains a major challenge to isolate internalized proteins to study their intracellular trafficking without confounding signal from remaining surface pools. Traditionally, fluorescent quenchers, such as Trypan Blue, have been added to the medium to mask fluorescent signals from the extracellular space,^18^ however, leaving fluorophores in place that may contribute to background. Alternatively, pH-sensitive markers like the fluorescent protein pHluorin or small molecules like the pH indicator Oregon Green have been used for visualizing exo- and endocytosis. However, these methods have limitations. The spectral characteristics of pHluorin are weak, limiting its utility in high-resolution techniques such as STED microscopy or PALM.^19^ Similarly, Oregon Green lacks selectivity as it does not bind to any protein, further hindering its effectiveness.^20^ Another classic strategy involves labeling the protein of interest with an antibody and then stripping any remaining antibodies from the surface.^21,22^ While effective, these techniques have limitations, including the size of the probes and the time required for the process.

Recent advancements have led to the development of cleavable probes, which offer several advantages over traditional methods, including smaller probe size and faster processing times. By placing a disulfide bond as a linker between the substrate and the dye, surface-remaining proteins can be cleaved of their labels by cell-impermeant reducing agents. Together, this facilitates precise visualization and quantitative measurements of endocytosis and recycling dynamics of internalized protein pools with drastically reduced background compared to strategies without a cleavable linker.^15,23^ With a limited repertoire of colors and tags to date, typically only one protein has been addressable at a time with this technique, making it difficult to observe multiple proteins and protein complexes in the same experiment. A similar argument can be made for BRET-based assays,^24^ where broad spectra make it difficult to color-multiplex in order to assess multiple proteins in the same preparation. To overcome these limitations, we synthesized four novel probes in the red and far-red spectral region that selectively bind to either the SNAP-tag or the Halo-tag, bear a disulfide bridge, and thus are rapidly and irreversibly cleavable in live cells *via* the impermeable reducing agent sodium 2-mercaptoethane sulfonate (MESNA). We characterized these molecules *in vitro* and in live cell settings, and investigated how they can be used as tool to simultaneously visualize and co-localize different metabotropic glutamate receptor (mGluR) heterodimers and class C G protein-coupled receptors (GPCRs). We further probe the internalization behaviour of two therapeutically relevant, co-expressed class B GPCRs, *i.e.* glucose-dependent insulinotropic polypeptide receptor (GIPR) and glucagon-like peptide-1 receptor (GLP1R) when stimulated with different (co-)agonists. Given the simultaneous nature of our measurements, we are able to observe co-internalization, and find evidence of receptor crosstalk.

## Results and discussion

**Fig. 1:**
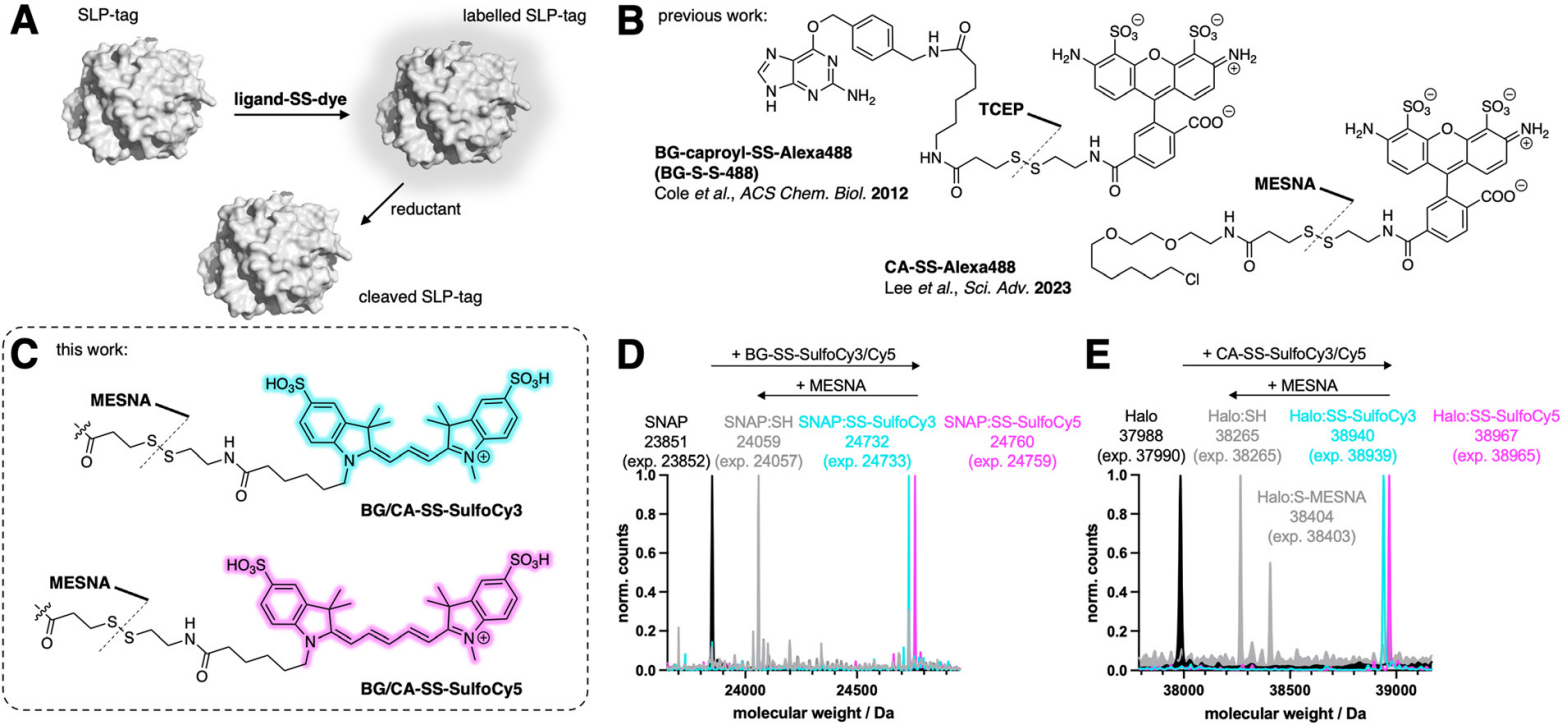
Fluorescent dyes for self-labelling protein (SLP) tags that are reductively cleavable. (A) Schematic of surface labelling with disulfide-containing probe and cleavage *via* a reducing agent. (B) Previous approaches for releasable self-labelling fluorophores: BG-caproyl-SS-Alexa488 cleaved with TCEP and CA-SS-Alexa488 cleaved with MESNA. (C) Structures of the four novel probes and approach for cleavage using MESNA. (D) Deconvoluted masses of: SNAP-tag protein (black), SNAP-tag protein incubated 1 h with 4-fold excess BG-SS-SulfoCy3 (teal), or BG-SS-SulfoCy5 (purple), 30 min after treatment with 100 mM MESNA (grey). (E) As for D, but with Halo-tag protein (black), and CA-SS-SulfoCy3 (teal), or CA-SS-SulfoCy5 (purple).

We aimed to design, synthesize, and evaluate four distinct probes for labeling target proteins fused to a self-labelling protein tag (SLP-tag). All of our probes featured a disulfide linker connecting the fluorophore to the SLP-tag substrate, enabling reductive cleavage of the fluorophore (Fig. 1A). Prior to our work, different strategies were employed by (i) utilizing a probe containing BG for the SNAP-tag, connected to the fluorescent AlexaFluor488 through both a caproyl linker and a disulfide linker, and (ii) employing a probe containing a CA for the Halo-tag, also linked to AlexaFluor488 *via* a disulfide bond. The disulfide bond cleavage in these previous designs was accomplished using the reducing agent tris(2-carboxyethyl)phosphine hydrochloride (TCEP) by Cole *et al.*, while sodium 2-mercaptoethane sulfonate (MESNA) was employed by Lee *et al.* (Fig. 1B). Other studies have used this strategy to label the SNAP-tag with a BG-SS-Lumi4-Tb chromophore for TR-FRET studies.^25–27^ Our primary objective was to expand the range of cleavable SLP-tag substrates by developing two orthogonally labelable probes in the red and far-red, since in these regimes less autofluorescence is observed. Using two highly hydrophilic fluorophores, SulfoCy3 and SulfoCy5, render the molecules cell impermeable. To connect these fluorophores to labeling moieties, we utilized a short disulfide bridge to attach them to either BG or CA, revealing that the linker length (omitting the caproyl unit in BG-SS-Alexa488) is sufficient. Consequently, we successfully synthesized four distinct probes: BG-SS-SulfoCy3, BG-SS-SulfoCy5, CA-SS-SulfoCy3, and CA-SS-SulfoCy5 (Fig. 1C and Fig. S2, ESI†). To evaluate the binding of these newly developed probes to the respective tag, we conducted *in vitro* labeling experiments using all four probes in conjunction with the recombinantly expressed and purified self-labeling tag.^28^ Additionally, we assessed the labeling efficiency both before and after treatment with MESNA using full-length protein mass spectrometry (Fig. 1D, E), demonstrating that a 4-fold excess of each probe quantitatively labeled the respective protein, and, complete cleavage of the probes was achieved within 30 minutes using an excess of MESNA (100 mM), adhering to the concentration used in previous studies (*vide supra*).

**Fig. 2:**
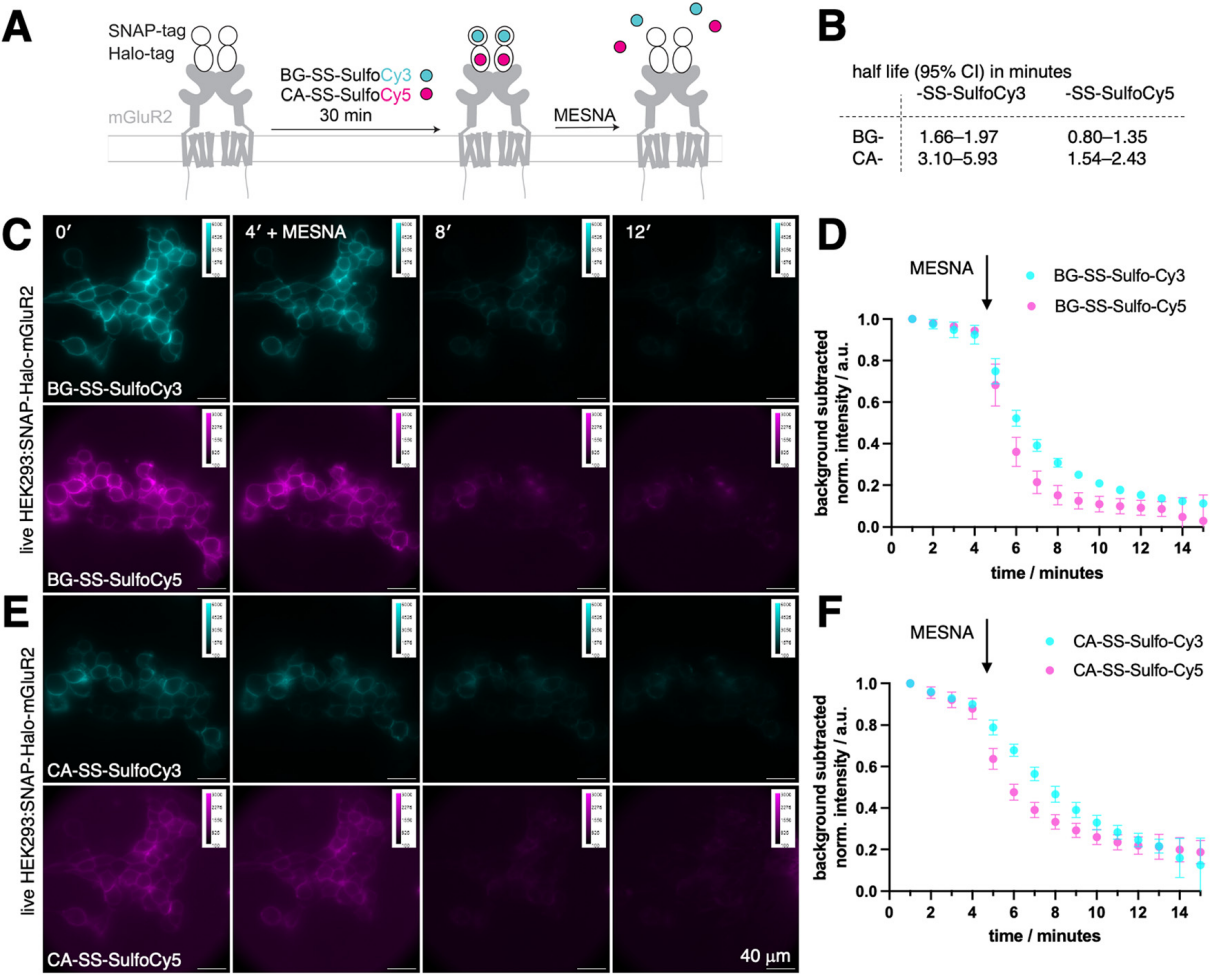
Time-dependence measurements of cleavable fluorescent dyes in cells. (A) Schematic representation of the experiment involving SNAP-Halo-mGluR2 transfected receptors binding to the respective probes, followed by cleavage with MESNA. (B) Half-life of BG-SS-SulfoCy3, BG-SS-SulfoCy5, CA-SS-SulfoCy3, and CA-SS-SulfoCy5 before binding to the respective SLP in minutes. 5 images analyzed. (C) Fluorescent images of SNAP-Halo-mGluR2 transfected HEK293 cells after incubation with BG-SS-SulfoCy3 and BG-SS-SulfoCy5 and after treatment with MESNA after 4 minutes. (D) Normalized integrated density of BG-SS-SulfoCy3 and BG-SS-SulfoCy5 over a 15-minute interval with MESNA added after 4 minutes reveals cleavage of the fluorophores. *n* = 5 images; mean ± SD. (E) As for C, but with CA-SS-SulfoCy3 and CA-SS-SulfoCy5. (F) As for D, but with CA-SS-SulfoCy3 and CA-SS-SulfoCy5.

To evaluate the effectiveness of our probes in a cellular environment, we conducted widefield imaging using live HEK293 cells that were transfected with a SNAP-Halo-mGluR2 coding plasmid. Generally, fusing proteins with tags that represent folded domains may hamper their endogenous dynamic behaviour, especially for smaller proteins and peptides. However, it has been shown in multiple studies, for mGluR subtypes^29–32^ as well as incretin GPCRs,^33–37^ that *N*-terminal SLP-tagging does not influence native ligand affinity and downstream signalling (*e.g.* cAMP generation or GIRK channel activation), including endocytotic uptake. For this reasons, any newly tagged protein needs to undergo careful comparison to its wild-type for validation, if ligand binding and subsequent signalling is perturbed. Titration series with ligands in the case of cell surface receptors may for instance be assessed by electrophysiology (for ion channels), second messenger generation (for GPCRs) and Western blotting of intracellular phosphorylation (receptor linked-enzymes). When expressed, metabotropic glutamate receptor 2 (mGluR2), a family C GPCR that is involved in a variety of neurophysiological processes,^38–40^ is trafficked to the cell surface, where it *N*-terminally exposes both SNAP and a Halo-tags. As such, intra and extracellular proteins pools are available as a testbed, and since the construct is equipped with two bioorthogonal self-labelling proteins, we could test two colors in parallel.^39,40^ Transiently transfected cells were incubated for 30-minutes with a combination of either BG-SS-SulfoCy3 and CA-SS-SulfoCy5, or BG-SS-SulfoCy5 and CA-SS-SulfoCy3, resulting in labeling localised to the cell surface (Fig. 2A). Upon addition of MESNA (100 mM in fluorobrite, pH = 7.2 at 37 °C and 5% CO_2_) to strip the fluorophore from the cell surface, we observed the cleavage of each probe in real time. As expected, with MESNA treatment, there was a 80–90% drop in fluorescence intensity. To analyze the kinetics of stripping dyes from the surface, we took the mean of the full image integrated density and corrected it by subtracting the mean integrated density from regions of interest, where no cells are present. The resulting curve was fitted mono-exponentially from minute 5 on, and some bleaching was detectable in the first 4 frames. Interestingly, stripping from SNAP was significantly faster compared to Halo (Fig. S1, ESI†), and the Cy5 conjugates were cleaved more rapidly than Cy3 (Fig. S1, ESI†). Specifically, we obtained the values for SNAP:SS-SulfoCy3 (*t*_1/2_ = 1.66–1.97 min), SNAP:SS-SulfoCy5 (*t*_1/2_ = 0.80–1.35 min), Halo:SS-SulfoCy3 (*t*_1/2_ = 3.10–5.93 min), and Halo:SS-SulfoCy5 (*t*_1/2_ = 1.54–2.43 min) by integrating the signal intensity of the complete image (Fig. 2B). Plateaus were reached for SNAP (Fig. 2C and D) and Halo (Fig. 2E and F) within 6 minutes. The difference in kinetics remains speculative, and may be attributed to the disparity in charge (*e.g.* sulfonates in Cy5 are more separated and thereby do not repell MESNA as much) or the accessibility of the disulfide bridge (*e.g.* by different exposure due to dye-protein interactions). Notably, the integrated fluorescence intensity in these experiments does not reach 0, presumably as a result from freely diffusing, cleaved dyes that contribute to the measured signal through out-of-focus light, and from a minor degree of constitutive receptor internalization that occurs during labelling.

**Fig. 3:**
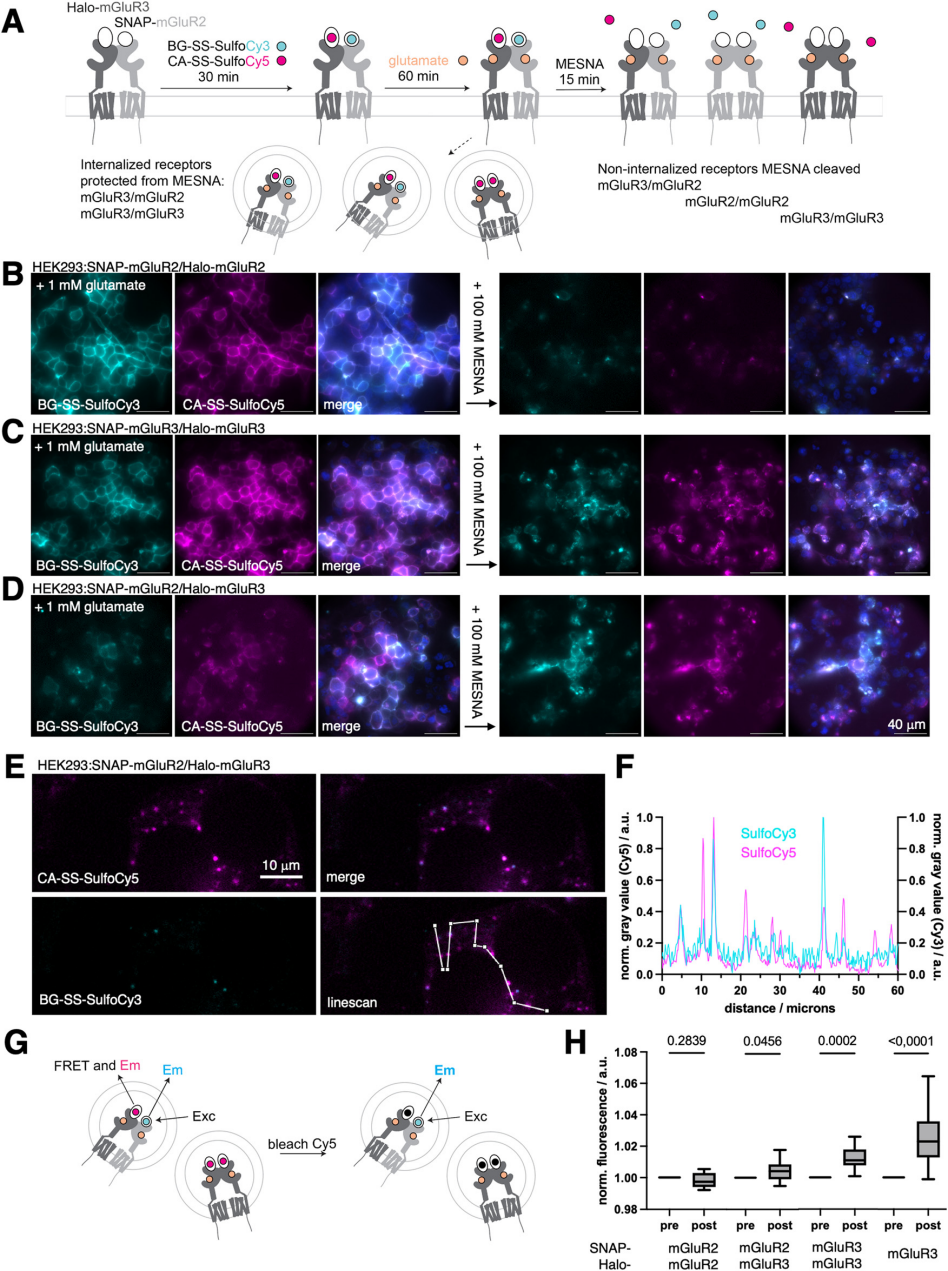
Internalization experiments of cleavable fluorescent dyes in cells. (A) Schematic representation of the experiment: SNAP- and Halo-mGluR bind to the respective probes, glutamate leads to internalization of mGluR3 containing receptor dimers, addition of MESNA cleaves fluorophores of remaining extracellular receptors. (B) Widefield imaging of live cells co-expressing SNAP-mGluR2:SS-SulfoCy3 and Halo-mGluR2:SS-SulfoCy5 after activation with 1 mM glutamate, and after additional 100 mM MESNA treatment. (C) As for B, but with SNAP-mGluR3:SS-SulfoCy3 and Halo-mGluR3:SS-SulfoCy5. (D) As for B but with SNAP-mGluR2:SS-SulfoCy3 and Halo-mGluR3:SS-SulfoCy5. (E) As for D, but with confocal imaging in fixed cells, and including linescan. (F) Line scan profiles from E reveal receptor colocalization in intracellular compartments. (G) Schematic representation of FRET experiment to measure Cy3 emission pre and post Cy5 bleaching, with enhanced donor signal post if within Förster radius (H) FRET measurements for SNAP-mGluR2/Halo-mGluR2, SNAP-mGluR2/Halo-mGluR3 and SNAP-mGluR3/Halo-mGluR3, with SNAP-Halo-mGluR3 serving as a positive control. Pre and post attributes acceptor bleaching. Min to max box and whiskers (*n* = 10 images), paired students *t*-test.

With these critical parameters in hand, we aimed to confirm the orthogonality of our dyes in the same experiment before and after endocytosis. An ideal testbed for assessing crosstalk beween receptors are the group II mGluRs, mGluR2 and mGluR3, as these receptor subtypes form both homo- and hetero-dimers.^29,30,41^ Using live cell imaging and single-molecule pull-down assays (SiMPull), we recently found evidence that mGluR3 homodimers and mGluR2/3 heterodimers, but not mGluR2 homodimers, undergo beta-arrestin-mediated endocytosis.^14,42^ However, in our prior study live cell imaging was done with the aforementioned CA-SS-Alexa488 probe, and as such, only one receptor subtype could be addressed at a time, making our measurements of intracellular heterodimers indirect. To further test the working model that mGluR2 homodimers do not internalize upon glutamate stimulation, while mGluR2/3 heterodimers and mGluR3 homodimers do (Fig. 3A), combinations of SNAP-mGluR2/Halo-mGluR2, SNAP-mGluR3/Halo-mGluR3, or SNAP-mGluR2/Halo-mGluR3 were transfected in HEK293 cells, before treatment with BG-SS-SulfoCy3 and CA-SS-SulfoCy5. After fluorophore labeling and washing, saturating (1 mM) glutamate was added for 60 min to drive receptor activation and internalization. As expected, HEK293 cells co-expressing SNAP-mGluR2/Halo-mGluR2 + glutamate, exhibited fluorescence signals exclusively on the cell surface for both fluorophores. Upon the addition of 100 mM MESNA, we observed very dim remaining signals (Cy3: 4 ± 2%; Cy5: 6 ± 3%, Fig. S2A, ESI†), consistent with a lack of internalization of mGluR2 homodimers (Fig. 3B). We applied the same protocol to cells expressing SNAP-mGluR3/Halo-mGluR3, and observed clear intracellular fluorescence both pre- and post-MESNA, indicating endocytosis of mGluR3 homodimers (Fig. 3C) (post MESNA signal Cy3: 67 ± 5%; Cy5: 43 ± 30%, Fig. S2A, ESI†). Similar results were obtained in cells expressing SNAP-mGluR2/Halo-mGluR3 after incubation with glutamate (Fig. 3D), further supporting the internalization of mGluR2/3 heterodimers (post MESNA signal Cy3: 44 ± 17%; Cy5: 52 ± 20%, Fig. S2A, ESI†). Having confirmed that the internalization propensity of mGluR2 is dramatically enhanced by co-expression of mGluR3, we aimed to quantify intracellular dimer pools by confocal microscopy in fixed specimens of HEK293:SNAP-mGluR2/Halo-mGluR3 and HEK293:SNAP-mGluR3/Halo-mGluR3 (Fig. S2B and C, ESI†), showcasing the power of dual color labelling and stripping. The degree of colocalization was assessed using Pearson's colocalization analysis, which demonstrated a more pronounced correlation of mGluR3 homodimers over mGluR2/mGluR3 heterodimers (Fig. S2D, ESI†). This is intuitive, since (i) the transfection of two separate constructs would also yield homodimers, of which mGluR2 will not internalize, (ii) most intracellular mGluR2 fluorescence co-localize with mGluR3, which (iii) is consistent with mGluR3 homo and heterodimers present. Providing further data for heteroassemblies, we performed line scan analysis (Fig. 3E) that show spatial overlap and co-localization of the two receptors (Fig. 3F). Importantly, we were able to measure dye communication *via* FRET by acceptor bleaching post MESNA treatment (Fig. 3G), which further demonstrates that internalized receptors are in close proximity (<10 nm) (Fig. 3H). Naturally, after MESNA treatment no FRET was detectable for SNAP-mGluR2/Halo-mGluR2 transfected cells while the propensity increased stepwise for SNAP-mGluR2/Halo-mGluR3 and SNAP-mGluR3/Halo-mGluR3, with SNAP-Halo-mGluR3 serving as a positive control. We do not anticipate cleavage by endogenous, reduced glutathione (GSH), since the environment in healthy endo- to lysosomes stays oxidative.^43^ In addition to this, if the dye is hypothetically cleaved when residing within the cells, it may not escape its compartment due to the charged nature of SulfoCy3/5 used in this study. Given confocal resolution, this would still account for accurate co-internalization, with the caveat that FRET is not observable anymore. Importantly, we show that high FRET signals stemming from intracellular sites can be obtained when expressing a SNAP-Halo-mGluR3 fusion construct, supporting the low probability of dye cleavage. Of note, other cleaving may be implemented to circumvent such issues if they may appear, for instance by using 1-(4,4-dimethyl-2,6-dioxocyclohex-1-ylidene)ethyl (Dde) and hydroxylamine or azobenzene and dithionite as a cleaving pair.^44^ Another possibility may be the incorporation of a proteolytic peptide sequence (*e.g.* HRV 3C, TEV) in accordance with their specific proteases.^45^

Having set the stage for the ability of cleavable dyes to simultaneously interrogate multiple GPCR subtypes, we aimed to employ our probes to investigate the relationship between agonist-induced internalization of the glucose-dependent insulinotropic polypeptide receptor (GIPR) and the glucagon-like peptide-1 receptor (GLP1R). In pancreatic islets, GLP1R and GIPR are co-expressed in beta cells, where they serve to potentiate insulin secretion to regulate blood glucose after a meal.^46^ Both receptors are also found in the central nervous system (CNS) where they regulate appetite, and whilst the CNS distribution of GLP1R and GIPR is mainly distinct, a small population of anorectic neurons is thought to co-express both receptors.^47^ Combinational targeting of GLP1R and GIPR has garnered significant attention, with tirzepatide (TZP), a dual GIPR/GLP1R agonist and new player for the treatment of type 2 diabetes and obesity, showing impressive results. The dual targeting approach of TZP is believed to produce synergistic effects on insulin secretion, glycemic control, and weight reduction, and reduced side effects.^48^ TZP is also of interest because of its unusual “biased” GLP1R pharmacology, characterized by markedly reduced beta-arrestin recruitment and endocytosis compared to native GLP1.^49^

**Fig. 4:**
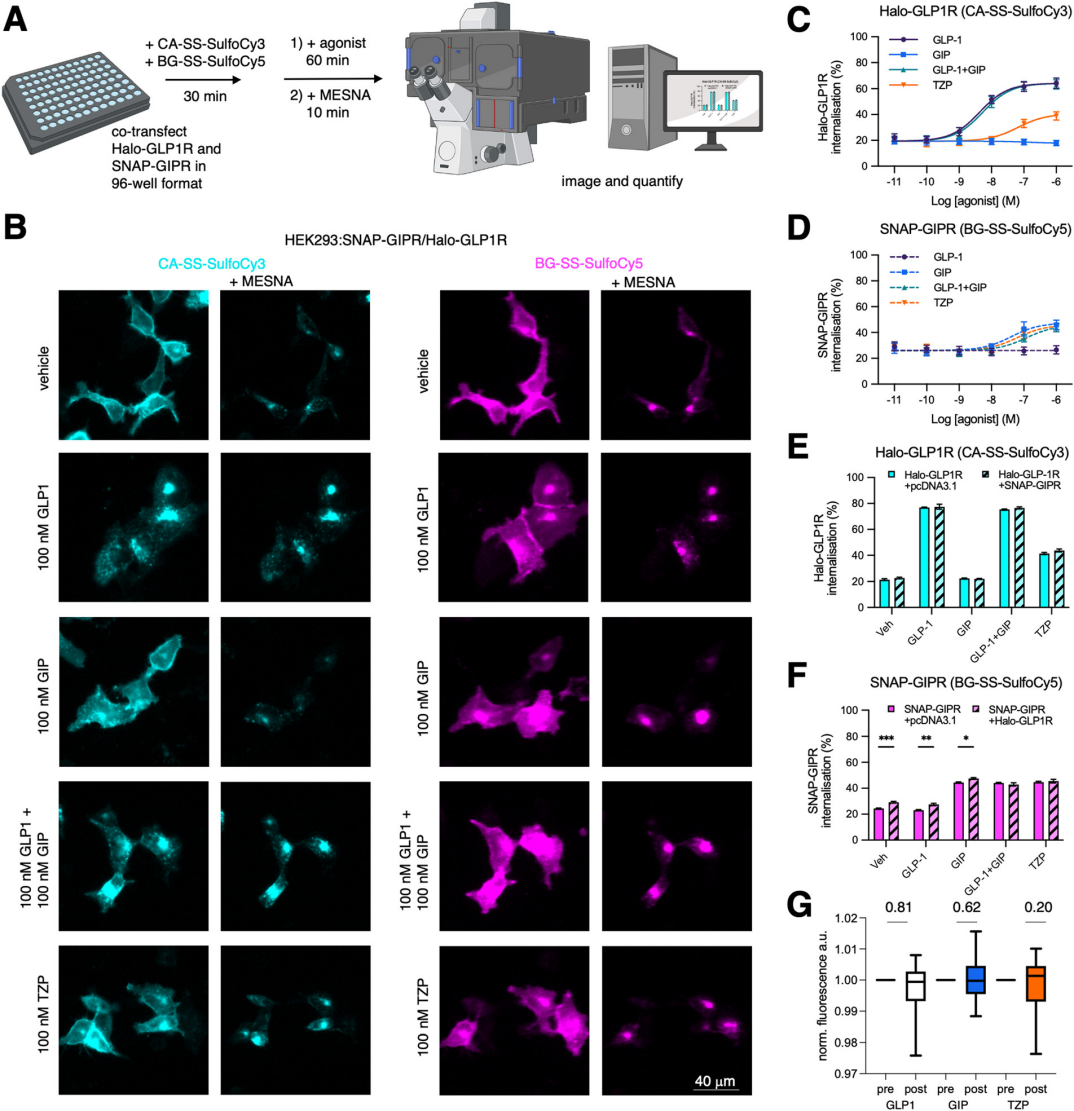
High-throughput experiments on internalization and colocalization of two receptors dependent on agonist. (A) Schematic representation of the experiment: Halo-GLP1R and SNAP-GIPR co-transfected cells are treated with cleavable fluorophores, treatment with agonist leads to internalization of receptors, addition of MESNA cleaves fluorophores of remaining extracellular receptors, remaining fluorophores are imaged and quantified. Image created with biorender.com (B) Widefield imaging of live cells co-expressing SNAP-GIPR and Halo-GLP1R after treatment with CA-SS-SulfoCy3 and BG-SS-SulfoCy5 and respective agonist, before and after treatment with MESNA. (C) and (D) Dose-dependent internalization curves of different agonists for Halo-GLP-1R (C), and SNAP-GIPR (D) (*n* = 6 repeats). (E) and (F) Quantification of internalization after treatment with respective agonist of Halo-GLP1R with and without co-expressed SNAP-GIPR (E), and SNAP-GIPR with and without co-expressed Halo-GLP1R (F) (*n* = 4 repeats). (G) FRET analysis of receptor internalization and proximity shows no increase in FRET efficiency. Min to max box and whiskers (*n* = 10–16 images) paired students *t*-test.

To investigate agonist-induced GLP1R and GIPR internalisation responses, adherent HEK293 (AD293) cells were co-transfected with Halo-GLP1R and SNAP-GIPR in a 96 well plate. After labelling with CA-SS-SulfoCy3 and BG-SS-SulfoCy5 for 30 min, agonist treatment was performed for 60 min, before imaging of multiple fields-of-view before and after application of MESNA using an automated microscope (Fig. 4A and B). This high throughput approach allows quantification of both internalized receptor fractions at several concentrations, allowing us to produce potency estimates for GLP1 (pEC_50_ 95% CI = 8.61–8.13 at GLP1R), GIP (7.97–6.84 at GIPR), equimolar co-applied GLP1 + GIP (8.50–8.06 at GLP1R, 7.67–5.82 at GIPR), and TZP (7.76–6.68 at GLP1R, 7.89–6.54 at GIPR) (Fig. 4C and D). We note also that both receptors show a constitutive internalization rate of ∼20–30% per hour in the absence of agonist, that maximal internalization with the respective native ligands is reduced with GIPR compared to GLP1R as previously shown,^50^ and finally that TZP has a reduced maximal GLP1R internalization response compared to native GLP1, in keeping with previous data.^49^

We then compared if co-expression leads to altered internalization levels of the respective receptor. For this experiment, we treated cells co-expressing Halo-GLP1R and SNAP-GIPR or each receptor individually with an empty pcDNA3.1 vector as transfection control. While our experimental findings indicate that the presence of the co-expressed SNAP-GIPR did not have any discernible impact on the behavior of Halo-GLP1R with any agonist (Fig. 4E), we observed a moderate but consistent increase in SNAP-GIPR, both constitutively and when treated with either GIP or GLP-1 alone (but not with both agonists together or the co-agonist TZP) (Fig. 4F). Since we did not observe an increase in Halo-GLP1R internalization when SNAP-GIPR was co-expressed, we conclude that the internalization of Halo-GLP1-R is independent of SNAP-GIPR. Conversely, it appears that there is crosstalk between Halo-GLP1R and SNAP-GIPR regarding the internalization of SNAP-GIPR. As we did for mGluR2 dimers, we also tested GLP1R/GIPR *via* FRET measurements once internalized (Fig. 4G). The Förster radius of Cy3/Cy5 has been determined to be between 5–6 nm, and energy transfer is not observable when crossing a distance further than 10 nm. This indicates that crosstalk is unlikely a consequence of direct GLP1R/GIPR interaction or heterodimerization, given the size of the receptors ectodomains (∼3 × 3 × 4 nm). Although dye and SLP orientation influencing κ^2^ may play a role, this points to non-mutual influence of receptor trafficking occurring at the plasmalemma.

## Summary

We have successfully developed four new releasable probes that fluoresce in the red- and far-red regime. To evaluate their binding and cleavage efficacy, we conducted both *in vitro* mass spectrometric and and in cell microscopic imaging. Additionally, we demonstrated that all probes can be cleaved within minutes using excess MESNA, which is favorably on the time scale of endocytosis. Validating our approach, we applied all probes to mGluRs and observed internalization and intracellular colocalization in live cells for both mGluR2/mGluR3 and mGluR3/mGluR3 assemblies when treated with glutamate. We also highlight the power of our approach by interrogating the internalization behaviour of GIPR and GLP1R, two main targets in the treatment of diabetes and obesity. In a 96-well plate assay to accelerate throughput, we determined dose-dependent internalization of co-expressed receptors towards various key agonists, *i.e.* GLP1, GIP and tirzepatide. Excitingly, we identified crosstalk between these two class B GPCRs, emphasizing the potential of our novel probes for studying endocytosis mediated trafficking. With higher-throughput agonist quantification of two receptors at the same time, we anticipate this method to be adoptable for multiplexible drug screening of cell surface proteins that undergo internalization.

## Materials and methods

Chemical synthesis and characterization, measurements of photophysical and kinetic parameters, and procedures in cell culture, molecular biology and imaging are reported in the ESI.† Further information and requests for resources and reagents should be directed to and will be fulfilled upon availability by the lead contact, Johannes Broichhagen (broichhagen@fmp-berlin.de)

## Author contributions

KR performed chemical synthesis and characterization. RB and BJ performed cell experiments. JL and JB conceived and supervised the study. KR, JL, BJ and JB wrote the manuscript with input from all authors.

## Conflicts of interest

The authors declare no competing interests.

## Supplementary Material

CB-006-D4CB00209A-s001

## Data Availability

The data supporting this article have been included as part of the ESI.†
