# Supplementary material for: Red and far-red cleavable fluorescent dyes for self-labelling enzyme protein tagging and interrogation of GPCR co-internalization
Source: RSC Chem Biol. 2024 Nov 18;6(1):11–20. doi: 10.1039/d4cb00209a (PMC11599839; doi:10.1039/d4cb00209a)
Supplement: CB-006-D4CB00209A-s001 [file CB-006-D4CB00209A-s001.pdf]

Supplementary Information (SI) for RSC Chemical Biology.
This journal is © The Royal Society of Chemistry 2024

Supporting Information for

Red and far-red cleavable fluorescent dyes for self-labelling enzyme protein tagging and interrogation of GPCR co-internalization

Kilian Roßmann[1], Ramona Birke[1], Joshua Levitz[2], Ben Jones[3], and Johannes Broichhagen[1,*]

[1] Leibniz-Forschungsinstitut für Molekulare Pharmakologie (FMP), 13125 Berlin, Germany.

[2] Department of Biochemistry, Weill Cornell Medicine, New York, NY, USA.

[3] Section of Endocrinology and Investigative Medicine, Department of Metabolism, Digestion and Reproduction, Imperial College London, London W12 0NN, UK

* Correspondence should be addressed to:
broichhagen@fmp-berlin.de

Contents

1 Methods 4

1.1 Culture, transfection and staining 4

1.2 Live Imaging 4

1.3 Fixed imaging 5

1.4 Picture analysis 5

1.5 High-content assessment of GLP1R/GIPR internalization 5

2 General Chemistry 7

3 Synthesis 9

3.1 2-((*E*)-3-((*E*)-1-(6-((2-((2-carboxyethyl)disulfaneyl)ethyl)amino)-6-oxohexyl)-3,3-dimethyl-5-sulfoindolin-2-ylidene)prop-1-en-1-yl)-1,3,3-trimethyl-3*H*-indol-1-ium-5-sulfonate (2) 9

3.2 2-((1*E*,3*E*)-5-((*E*)-1-(6-((2-((2-carboxyethyl)disulfaneyl)ethyl)amino)-6-oxohexyl)-3,3-dimethyl-5-sulfoindolin-2-ylidene)penta-1,3-dien-1-yl)-1,3,3-trimethyl-3*H*-indol-1-ium-5-sulfonate (4) 10

3.3 2-((*E*)-3-((*E*)-1-(6-((2-((3-((4-(((2-amino-9*H*-purin-6-yl)oxy)methyl)benzyl)amino)-3-oxopropyl)disulfaneyl)ethyl)amino)-6-oxohexyl)-3,3-dimethyl-5-sulfoindolin-2-ylidene)prop-1-en-1-yl)-1,3,3-trimethyl-3*H*-indol-1-ium-5-sulfonate (BG-SS-SulfoCy3) 11

3.4 2-((1*E*,3*E*)-5-((*E*)-1-(6-((2-((3-((4-(((2-amino-9*H*-purin-6-yl)oxy)methyl)benzyl)amino)-3-oxopropyl)disulfaneyl)ethyl)amino)-6-oxohexyl)-3,3-dimethyl-5-sulfoindolin-2-ylidene)penta-1,3-dien-1-yl)-1,3,3-trimethyl-3*H*-indol-1-ium-5-sulfonate (BG-SS-SulfoCy5) 12

3.5 2-((*E*)-3-((*E*)-1-(27-chloro-6,14-dioxo-18,21-dioxa-10,11-dithia-7,15-diazaheptacosyl)-3,3-dimethyl-5-sulfoindolin-2-ylidene)prop-1-en-1-yl)-1,3,3-trimethyl-3*H*-indol-1-ium-5-sulfonate (CA-SS-SulfoCy3) 13

3.6 2-((1*E*,3*E*)-5-((*E*)-1-(6-((2-((3-((4-(((2-amino-9*H*-purin-6-yl)oxy)methyl)benzyl)amino)-3-oxopropyl)disulfaneyl)ethyl)amino)-6-oxohexyl)-3,3-dimethyl-5-sulfoindolin-2-ylidene)penta-1,3-dien-1-yl)-1,3,3-trimethyl-3*H*-indol-1-ium-5-sulfonate (CA-SS-SulfoCy5) 14

4 NMR-Spectra 15

4.1 2-((*E*)-3-((*E*)-1-(6-((2-((2-carboxyethyl)disulfaneyl)ethyl)amino)-6-oxohexyl)-3,3-dimethyl-5-sulfoindolin-2-ylidene)prop-1-en-1-yl)-1,3,3-trimethyl-3*H*-indol-1-ium-5-sulfonate (2) 15

4.2 2-((1*E*,3*E*)-5-((*E*)-1-(6-((2-((2-carboxyethyl)disulfaneyl)ethyl)amino)-6-oxohexyl)-3,3-dimethyl-5-sulfoindolin-2-ylidene)penta-1,3-dien-1-yl)-1,3,3-trimethyl-3*H*-indol-1-ium-5-sulfonate (4) 16

4.3 2-((*E*)-3-((*E*)-1-(6-((2-((3-((4-(((2-amino-9*H*-purin-6-yl)oxy)methyl)benzyl)amino)-3-oxopropyl)disulfaneyl)ethyl)amino)-6-oxohexyl)-3,3-dimethyl-5-sulfoindolin-2-ylidene)prop-1-en-1-yl)-1,3,3-trimethyl-3*H*-indol-1-ium-5-sulfonate (BG-SS-SulfoCy3) 17

4.4 2-((1*E*,3*E*)-5-((*E*)-1-(6-((2-((3-((4-(((2-amino-9*H*-purin-6-yl)oxy)methyl)benzyl)amino)-3-oxopropyl)disulfaneyl)ethyl)amino)-6-oxohexyl)-3,3-dimethyl-5-sulfoindolin-2-ylidene)penta-1,3-dien-1-yl)-1,3,3-trimethyl-3*H*-indol-1-ium-5-sulfonate (BG-SS-SulfoCy5) 18

4.5 2-((*E*)-3-((*E*)-1-(27-chloro-6,14-dioxo-18,21-dioxa-10,11-dithia-7,15-diazaheptacosyl)-3,3-dimethyl-5-sulfoindolin-2-ylidene)prop-1-en-1-yl)-1,3,3-trimethyl-3*H*-indol-1-ium-5-sulfonate (CA-SS-SulfoCy3) 19

4.6 2-((1*E*,3*E*)-5-((*E*)-1-(6-((2-((3-((4-(((2-amino-9*H*-purin-6-yl)oxy)methyl)benzyl)amino)-3-oxopropyl)disulfaneyl)ethyl)amino)-6-oxohexyl)-3,3-dimethyl-5-sulfoindolin-2-ylidene)penta-1,3-dien-1-yl)-1,3,3-trimethyl-3*H*-indol-1-ium-5-sulfonate (CA-SS-SulfoCy5) 20

5 References 21

1 Methods

1.1 Culture, transfection and staining

HEK293T cells were cultured in growth medium (DMEM, Glutamax, 4.5 g Glucose, 10% FCS, 1% PS; Invitrogen) at 37 °C and 5% CO_2. 50 000 cells per well were seeded on 8-well µL slides (Ibidi) previously coated with poly-L-lysine (Aldrich, mol wt 70 000–150 000). The next day, 400 ng DNA was transfected using 0.8 µL Jet Prime reagent in 40 µL Jet Prime buffer (VWR) per well/plasmid. Medium was exchanged against antibiotic-free media before the transfection mix was pipetted on the cells. After 4 hours incubation at 37 °C and 5% CO_2, medium was exchanged against growth media, and after an additional 24 hours, cells were stained. All cleavable dyes were used at a concentration of 500 nM with the addition of Hoechst 33342 at 1 µM, in growth medium at 37 °C and 5% CO_2 for 30 minutes. Afterwards cells were washed once in growth media.

1.2 Live Imaging

Imaged live in fluorobrite (Invitrogen) using an epifluorescence Nikon Ti-E microscope, equipped with pE4000 (cool LED), Penta Cube (AHF 66-615), 60× oil NA 1.49 (Apo TIRF Nikon) and imaged on a sCMOS camera (Prime 95B, Photometrics) operated by NIS Elements (Nikon). For excitation the following LED wavelengths were used: Hoechst – 405 nm, Cy3 – 550 nm, Cy5 – 635 nm.
For live cell imaging transfected cells were stained and then treated with 1 mM glutamate for 60 min, before the dyes were cleaved off using 100 mM MESNA for 10 minutes.

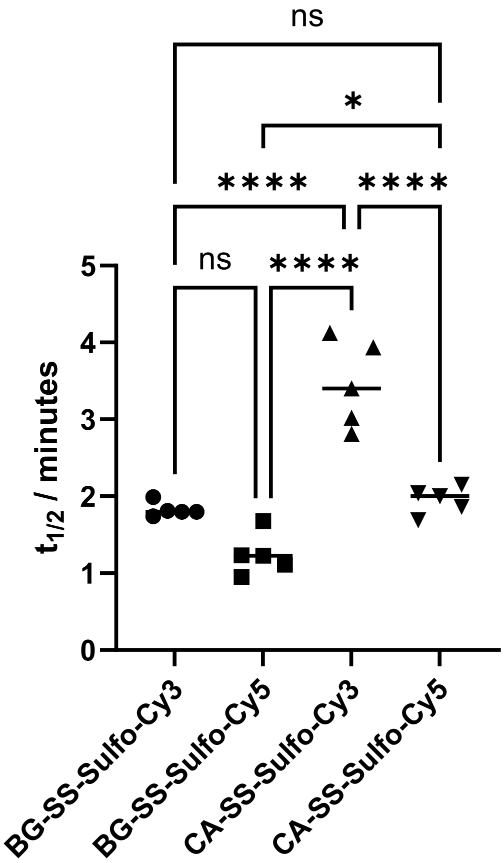

Supplemental Figure S1: Half-life after 100 mM treatment with Mesna. *n*=5. * indicates statistical significance (one-way ANOVA, *p<0.05, ****p<0.0001, ns = non-significant). Mean±SD.

1.3 Fixed imaging

Fixed cells on NIKON-CSU-X1 (Nikon Ti Eclipse with automatic stage and Perfect Focus System, equipped with Yokogawa spinning disk (CSU-X1, 1000 scan/s) and EMCCD Camera (AU-888, 13 um pixel) and laser: 405 (120 mW), 445 (100 mW), 488 (200 mW), 514 (100 mW), 561 (150 mW), 638 (200 mW) for fast confocal imaging, objectives: 20x air NA 0.75, 40x air NA 0.95, 60x oil NA 1.4, and field of view: 53 x 53µm (100x) up to 266 x 266 µm (20x)). For confocal imaging, transfected and stained cells were treated with 1 mM glutamate for 20 minutes, either fixed or treated with 100 mM MESNA for another 10 minutes and fixed afterwards. For fixation 2% PFA (Thermo Fisher) in PBS was used for 30 min at room temperature. Cells were washed 3 times in PBS and the reaction was quenched using 0.1 M glycine/0.1 NH4Cl in PBS for 10 min, cells were washed again 3 times and imaged in PBS.

1.4 Picture analysis

Integrated density was measured on 5 pictures for each cleavable dye for every frame using ImageJ, by drawing a region of interest (ROI) around transfected cell cluster, averaged and normalized (background levels from regions without cells were substracted).
Colocalization analysis were also performed using ImageJ and the Coloc2 function for 10 images for each condition and depicted as averaged, normalized Pearson`s value without threshold. For the control the Cy5 channel was rotated 90° to right. Line scans were drawn with the polygon selection in ImageJ and the grey values obtained were normalized and plotted against the distance.

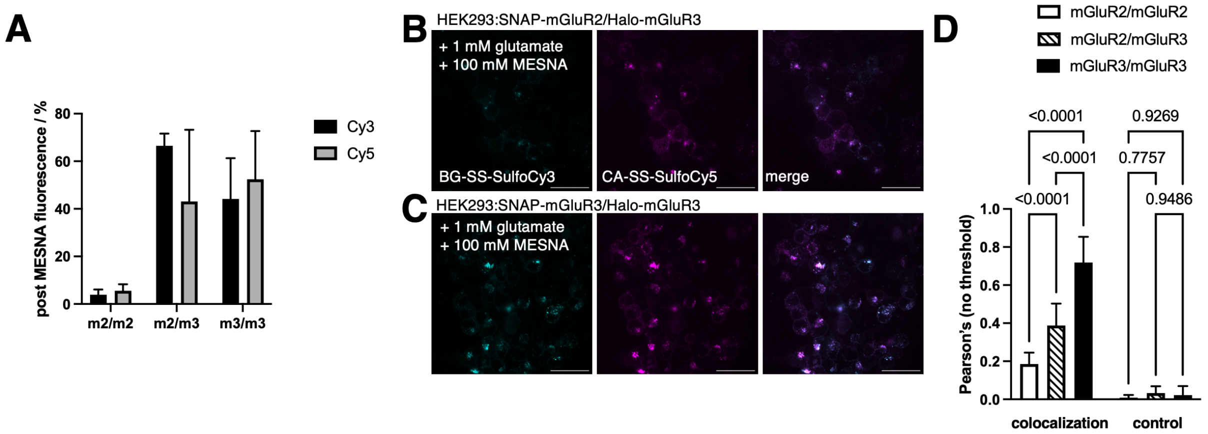

Supplemental Figure S2: (**A**) Mean fluorescence of images post MESNA with respect to pre MESNA, background subtracted. (**B**) Confocal imaging in fixed cells co-expressing SNAP-mGluR2:SS-SulfoCy3 and Halo-mGluR3:SS-SulfoCy5 after activation with 1 mM glutamate, and after additional 100 mM MESNA treatment. (**C**) As for A, but with SNAP-mGluR3:SS-SulfoCy3 and Halo-mGluR3:SS-SulfoCy5 (**D**) Pearson's colocalization analysis of respective mGluR. Control is using same images with acceptor channel rotated by 90°. One-way ANOVA, error bars show mean + SD.

1.5 High-content assessment of GLP1R/GIPR internalization

AD293 cells were reverse-transfected with 0.1 µg total DNA consisting of SNAP-GLP1R and Halo-GIPR vectors in a 2:1 ratio using Lipofectamine 2000 in 96-well, black, clear-bottomed plates coated with poly-D-lysine. 24 hours after transfection, cells were labelled using 0.5 µM BG-SS-Sulfo-Cy5 and CA-SS-Sulfo-Cy3 for 30 min at 37 °C in complete DMEM. After washing, agonists were applied in serum-free DMEM with 0.1% BSA to cover the indicated concentration range. After a 60-min incubation at 37 °C, cells were washed and placed in alkaline TNE buffer (pH 8.6) and the microplate was transferred to the microscope. Imaging was performed using a Nikon Ti-2E-based instrument with automated stage (ASI), LED and phase contrast transmitted illumination, image splitter (Cairn Optosplit III) and sCMOS camera (Photometrics

Iris 15). Between 9 and 15 fields-of-view were acquired for each well for both transmitted and phase contrast at wavelengths corresponding to Cy3 and Cy5 probes. After the baseline images for a well was completed, MESNA (final concentration = 100 mM) was added, and post-MESNA images were acquired after a 10 min delay. Image analysis was performed as previously described, with flat-field and background subtraction using BaSiC and cell segmentation using the phase contrast images via PHANTAST. Non-transfected wells were used to establish non-specific labelling, which was also subtracted. Specific signal from post-MESNA cell-containing regions (internalized receptor) was expressed relative to the corresponding pre-MESNA image (total receptor), providing a quantitative measurement of internalization in percentage terms.

2 General Chemistry

All chemical reagents and anhydrous solvents for synthesis were purchased from commercial suppliers (Roth, Broadpharm, lumiprobe, Sigma-Aldrich, TCI) and were used without further purification if not stated otherwise.

NMR spectra were recorded at 300 K in deuterated solvents on a Bruker AV-III spectrometer using a room- temperature 5 mm broadband probe equipped with one-axis self-shielded gradients and calibrated to residual solvent peaks ($^{1}H/^{13}C$ in ppm): DMSO-d_6 (2.50/40.00), MeOD-d4 (3.31/49.00). Multiplicities are abbreviated as follows: s = singlet, d = doublet, t = triplet, q = quartet, p = pentet, h = heptet, br = broad, m = multiplet. Coupling constants *J* are reported in Hz. Spectra are reported based on appearance, not on theoretical multiplicities derived from structural information.

UPLC-UV/Vis for purity assessment was performed on an Agilent 1260 Infinity II LC System equipped with Agilent SB-C18 column (1.8 µm, 2.1 × 50 mm). Buffer A: 0.1% FA in H_2O Buffer B: 0.1% FA acetonitrile. The typical gradient was from 10% B for 1.0 min → gradient to 95% B over 5 min → 95% B for 1.0 min with 0.45 mL/min flow. Retention times (t_R) are given in minutes (min). Chromatograms were imported into Graphpad Prism8 and purity was determined by calculating AUC ratios. LCMS traces were normalized from blank run via Agilent OpenLab software 1260.

Preparative or semi-preparative HPLC was performed on an Agilent 1260 Infinity II LC System equipped with columns as followed: preparative column –Reprospher 100 C18 columns (10 µm: 50 x 30 mm at 20 mL/min flow rate; semi-preparative column – 5 µm: 250 x 10 mm at 4 mL/min flow rate. Eluents A (0.1% TFA in H_2O) and B (0.1% TFA in MeCN) were applied as a linear gradient. Peak detection was performed at maximal absorbance wavelength.

For HRMS, samples were analyzed on Orbitrap Fusion mass spectrometer (Thermo Fisher Scientific). MS scans were acquired in a range of 350 to 1500 m/z. MS1 scans were acquired in the Orbitrap with a mass resolution of 120,000 with an AGC target value of 4e5 and 50 ms injection time. MS2 scans were acquired in the ion trap with an AGC target value of 1e4 and 35 ms injection time. Precursor ions with charge states 2-4 were isolated with an isolation window of 1.6 m/z and 40 sec dynamic exclusion. Precursor ions were fragmented using higher-energy collisional dissociation (HCD) with 30% normalized collision energy.

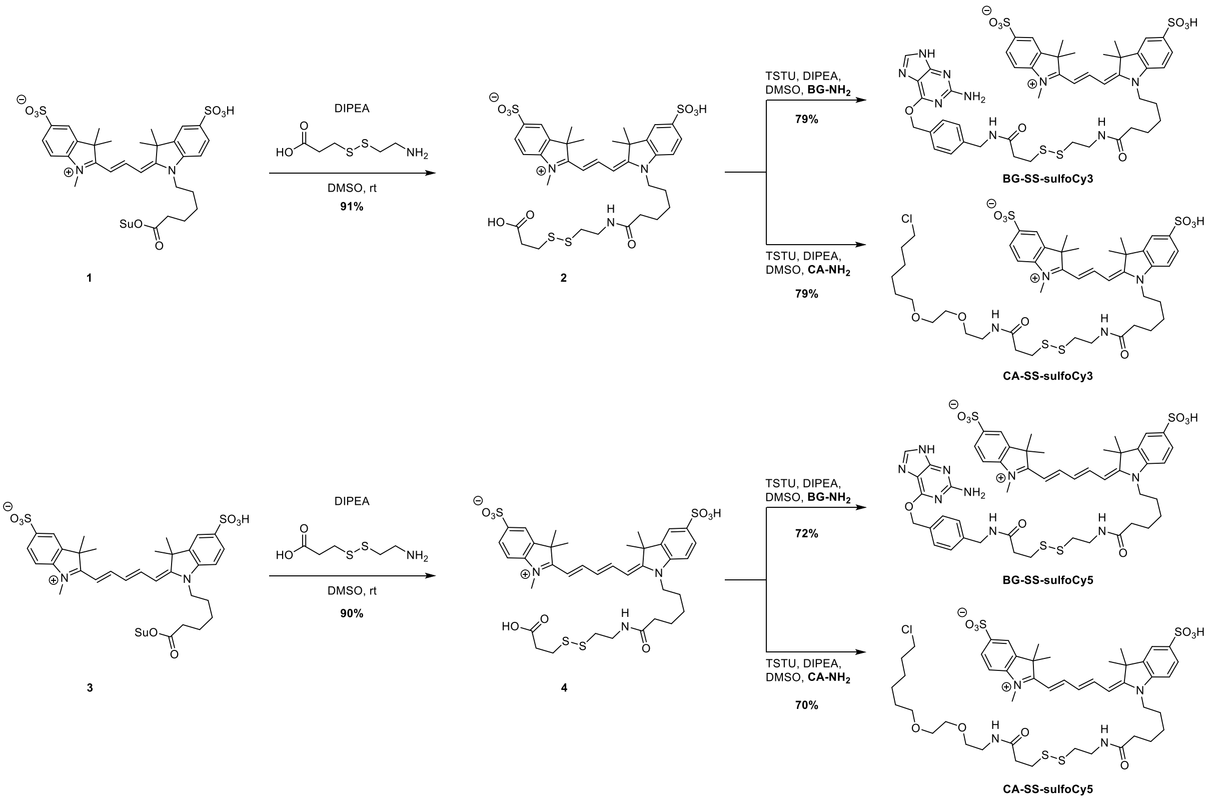

Supplemental Scheme 1: Chemical synthesis of cleavable probes.

3 Synthesis

3.1 2-((*E*)-3-((*E*)-1-(6-((2-((2-carboxyethyl)disulfaneyl)ethyl)amino)-6-oxohexyl)-3,3-dimethyl-5-sulfoindolin-2-ylidene)prop-1-en-1-yl)-1,3,3-trimethyl-3*H*-indol-1-ium-5-sulfonate (2)

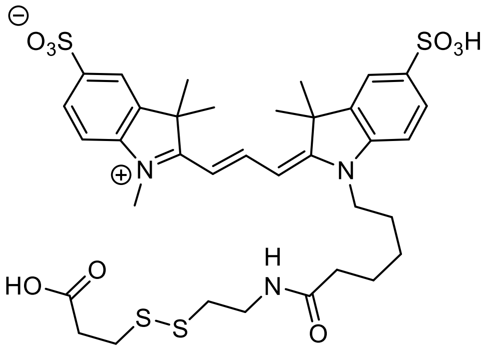

SulfoCy3-NHS (10.0 mg, 13.3 µmol, 1.0 equiv.) was dissolved in 1 mL DMSO, before DIPEA (9.3 µL, 53.2 µmol, 4.0 equiv.) and 3-((2-aminoethyl)disulfaneyl)propanoic acid (**1**) (2.7 mg, 14.6 µmol, 1.1 equiv.) were added. The reaction was stirred for 30 min at rt, before it was quenched by addition of glacial HOAc (20 µL). The reaction mixture was subjected to RP-HPLC to obtain 10.8 mg (12.1 µmol) of the desired compound as a red powder in 91% yield.

^{1}H NMR (600 MHz, DMSO-d$_6$) [ppm] = δ 8.36 (t, *J* = 13.5 Hz, 2H), 7.98 (t, *J* = 5.6 Hz, 1H), 7.81 (t, *J* = 1.9 Hz, 2H), 7.68 (t, *J* = 8.2 Hz, 2H), 7.39 (t, *J* = 7.8 Hz, 2H), 6.49 (dd, *J* = 13.4, 4.2 Hz, 2H), 4.11 (t, *J* = 7.4 Hz, 2H), 3.66 (s, 3H), 3.30 (q, *J* = 6.4 Hz, 2H), 2.87 (t, *J* = 7.0 Hz, 2H), 2.76 (t, *J* = 6.8 Hz, 2H), 2.61 (t, *J* = 7.0 Hz, 2H), 2.08 (t, *J* = 7.3 Hz, 2H), 1.71 (s, 12H), 1.57 (p, *J* = 7.5 Hz, 2H), 1.38 (p, *J* = 7.6 Hz, 2H).

^{13}C NMR (151 MHz, DMSO-d$_6$): *δ* [ppm] = 175.3, 174.5, 173.1, 172.5, 150.2, 146.4, 146.2, 143.0, 142.3, 140.5, 126.7, 126.6, 120.4, 120.3, 111.1, 103.8, 103.2, 49.4, 44.3, 38.3, 37.8, 35.5, 34.1, 33.6, 31.8, 29.5, 29.2, 27.9, 27.7, 27.2, 26.2, 25.3.

HRMS (ESI): calc. for $C_{35}H_{46}N_3O_9S_4^+$ $[M]^+$: 780.2111, found: 780.2113.

3.2 2-((1*E*,3*E*)-5-((*E*)-1-(6-((2-((2-carboxyethyl)disulfaneyl)ethyl)amino)-6-oxohexyl)-3,3-dimethyl-5-sulfoindolin-2-ylidene)penta-1,3-dien-1-yl)-1,3,3-trimethyl-3*H*-indol-1-ium-5-sulfonate (4)

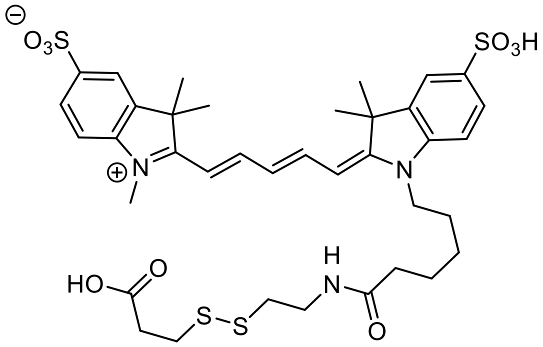

SulfoCy5-NHS (10.0 mg, 12.9 µmol, 1.0 equiv.) was dissolved in 1 mL DMSO, before DIPEA (9.0 µL, 51.6 µmol, 4.0 equiv.) and 3-((2-aminoethyl)disulfaneyl)propanoic acid (**3**) (2.6 mg, 14.2 µmol, 1.1 equiv.) were added. The reaction was stirred for 30 min at rt, before it was quenched by addition of glacial HOAc (20 µL). The reaction mixture was subjected to RP-HPLC to obtain 10.7 mg (11.6 µmol) of the desired compound as a blue powder in 90% yield.

^{1}H NMR (600 MHz, DMSO-d$_6$): *δ* [ppm] = 8.36 (t, *J* = 13.1 Hz, 2H), 7.96 (t, *J* = 5.6 Hz, 1H), 7.81 (s, 2H), 7.64 (t, *J* = 2.2 Hz, 2H), 7.31 (dd, *J* = 8.2, 5.8 Hz, 2H), 6.57 (t, *J* = 12.3 Hz, 1H), 6.29 (dd, *J* = 27.5 13.8, Hz, 2H), 4.08 (t, *J* = 7.2 Hz, 2H), 3.60 (s, 3H), 3.30 (q, *J* = 6.4 Hz, 2H), 2.88 (t, *J* = 7.0 Hz, 2H), 2.76 (t, *J* = 6.8 Hz, 2H), 2.62 (t, *J* = 7.0 Hz, 2H), 2.06 (t, *J* = 7.3 Hz, 2H), 1.70 (s, 12H), 1.55 (m, *J* = 7.4 Hz, 2H), 1.34 (m, *J* = 7.6 Hz, 2H).

^{13}C NMR (151 MHz, DMSO-d$_6$): *δ* [ppm] = 174.1, 173.3, 173.1, 172.5, 154.6, 145.8, 145.6, 143.1, 142.4, 140.9, 140.8, 126.5, 126.4, 126.1, 120.4, 120.3, 110.5, 110.5, 104.0, 103.8, 49.3, 43.8, 38.2, 37.7, 35.5, 34.1, 33.5, 31.6, 29.4, 29.1, 27.5, 27.3, 27.1, 26.1, 25.3.

HRMS (ESI): calc. for $C_{37}H_{48}N_3O_9S_4^+$ $[M]^+$: 806.2268, found: 806.2268.

3.3 2-((*E*)-3-((*E*)-1-(6-((2-((3-((4-(((2-amino-9*H*-purin-6-yl)oxy)methyl)benzyl)amino)-3-oxopropyl)disulfaneyl)ethyl)amino)-6-oxohexyl)-3,3-dimethyl-5-sulfoindolin-2-ylidene)prop-1-en-1-yl)-1,3,3-trimethyl-3*H*-indol-1-ium-5-sulfonate (BG-SS-SulfoCy3)

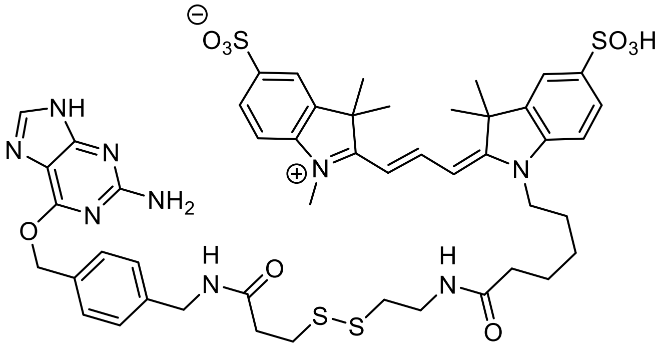

2 (5.4 mg, 6.1 µmol 1.0 equiv.) was dissolved in 1 mL DMSO, before DIPEA (4.1 µL, 24.4 µmol, 4.0 equiv.) and TSTU (2.8 mg, 9.2 µmol, 1.5 equiv.) were added. The reaction was stirred for 30 min at rt, before it was quenched by addition of glacial HOAc (20 µL). The reaction mixture was subjected to RP-HPLC. The product containing fractions were combined, lyophilized, and dissolved in 1 mL DMSO. DIPEA (4.1 µL, 24.4 µmol, 4.0 equiv.) and BG-NH_2 (2.0 mg, 7.32 µmol, 1.2 equiv.) were added and the reaction was stirred for 30 min at rt, before it was quenched by addition of glacial HOAc (20 µL). The reaction mixture was subjected to RP-HPLC to obtain 5.5 mg (4.8 µmol) of the desired compound as a red powder in 79% yield.

^{1}H NMR (600 MHz, DMSO-d$_6$): δ [ppm] = 8.49 (t, *J* = 5.9 Hz, 1H), 8.34 (t, *J* = 13.5 Hz, 1H), 7.97 (t, *J* = 5.6 Hz, 1H), 7.81 (s, 2H), 7.69 (ddd, *J* = 8.2, 3.7, 1.6 Hz, 2H), 7.49 (d, *J* = 8.1 Hz, 2H), 7.38 (dd, *J* = 11.2, 8.3 Hz, 2H), 7.29 (d, *J* = 8.1 Hz, 2H), 7.22 (q, *J* = 11.7 Hz, 1H), 6.48 (dd, *J* = 13.4, 4.7 Hz, 2H), 5.53 (s, 2H), 4.28 (d, *J* = 5.8 Hz, 2H), 4.10 (t, *J* = 7.2 Hz, 2H), 3.66 (s, 3H), 3.64-3.61 (m, 2H), 3.30 (q, *J* = 5.9 Hz, 1H), 2.92 (t, *J* = 7.1 Hz, 2H), 2.75 (t, *J* = 6.7 Hz, 2H), 2.54 (t, *J* = 7.2 Hz, 2H), 2.07 (t, *J* = 7.2 Hz, 2H), 1.70 (s, 12H), 1.57 (p, *J* = 7.0 Hz, 2H), 1.36 (p, *J* = 7.7 Hz, 2H).

^{13}C NMR (151 MHz, DMSO-d$_6$): δ [ppm] = 175.3, 174.4, 173.4, 172.5, 170.5 150.13, 146.3, 146.1, 143.0, 142.3, 140.5, 129.3, 127.7, 126.7, 126.6, 120.3, 120.2, 111.0, 103.8, 103.2, 69.7, 65.9, 63.0, 49.3, 44.2, 42.3, 38.2, 37.7, 35.4, 35.4, 34.4, 33.9, 32.0, 31.7, 29.4, 29.3, 29.1, 28.9, 27.9, 27.6, 27.1, 26.1, 25.3, 24.9.

HRMS (ESI): calc. for $C_{48}H_{58}N_9O_9S_4^+$ $[M]^+$: 1032.3235, found: 1032.3243.

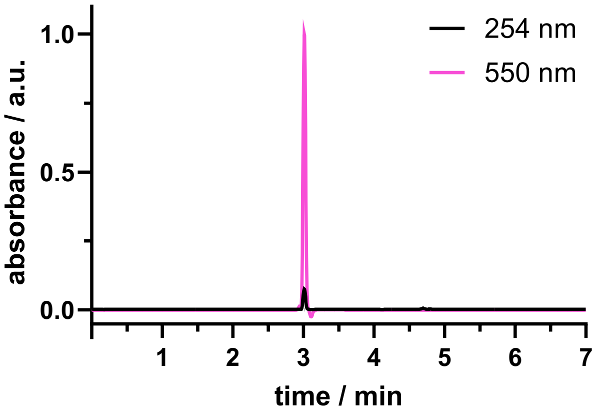

3.4 2-((1*E*,3*E*)-5-((*E*)-1-(6-((2-((3-((4-(((2-amino-9*H*-purin-6-yl)oxy)methyl)benzyl)amino)-3-oxopropyl)disulfaneyl)ethyl)amino)-6-oxohexyl)-3,3-dimethyl-5-sulfoindolin-2-ylidene)penta-1,3-dien-1-yl)-1,3,3-trimethyl-3*H*-indol-1-ium-5-sulfonate (BG-SS-SulfoCy5)

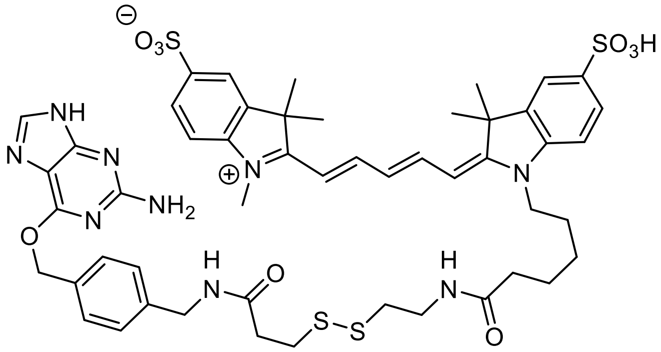

4 (5.3 mg, 5.8 µmol 1.0 equiv.) was dissolved in 1 mL DMSO, before DIPEA (4.0 µL, 23.2 µmol, 4.0 equiv.) and TSTU (2.6 mg, 8.7 µmol, 1.5 equiv.) were added. The reaction was stirred for 30 min at rt, before it was quenched by addition of glacial HOAc (20 µL). The reaction mixture was subjected to RP-HPLC. The product containing fractions were combined, lyophilized, and dissolved in 1 mL DMSO. DIPEA (4.0 µL, 23.2 µmol, 4.0 equiv.) and BG-NH_2 (1.9 mg, 7.0 µmol, 1.2 equiv.) were added. The reaction was stirred for 30 min at rt, before it was quenched by addition of glacial HOAc (20 µL). The reaction mixture was subjected to RP-HPLC to obtain 4.9 mg (4.2 µmol) of the desired compound as a blue powder in 72% yield.

^{1}H NMR (600 MHz, DMSO-d_6): *δ* [ppm] = 8.59 (s, 1H), 8.49 (t, *J* = 5.9 Hz, 1H), 8.35 (t, *J* = 13.0 Hz, 2H), 7.96 (t, *J* = 5.6 Hz, 1H), 7.82 (s, 2H), 7.67-7.63 (m, 2H), 7.49 (d, *J* = 8.0 Hz, 2H), 7.34-7.27 (m, 4H), 6.56 (t, *J* = 12.3 Hz, 1H), 6.28 (dd, *J* = 25.6, 13.8 Hz, 2H), 5.54 (s, 1H), 4.28 (d, *J* = 5.9 Hz, 2H), 4.08 (d, *J* = 6.8 Hz, 2H), 3.59 (s, 3H), 3.35 (m, *J* = 5.2 Hz, 2H), 3.30 (m, *J* = 6.1 Hz, 2H), 2.92 (t, *J* = 7.0 Hz, 2H), 2.75 (t, *J* = 6.7 Hz, 2H), 2.55 (t, *J* = 7.1 Hz, 2H), 2.05 (t, *J* = 4.8 Hz, 2H), 1.69 (s, 12H), 1.54 (p, *J* = 7.2 Hz, 2H), 1.33 (p, *J* = 7.5 Hz, 2H).

^{13}C NMR (151 MHz, DMSO-d_6): *δ* [ppm] = 174.1, 173.4, 173.3, 172.5, 170.5, 159.1, 154.6, 145.8, 145.5, 143.1, 142.5, 140.9, 140.8, 140.4, 134.3, 129.3, 127.7, 126.8, 126.5, 126.4, 126.1, 120.4, 120.3, 110.5, 104.0, 103.8, 69.7, 68.7, 65.9, 63.1, 49.3, 43.8, 42.3, 38.2, 37.7, 35.4, 35.4, 34.4, 33.9, 31.7, 29.4, 29.3, 29.1, 28.9, 27.5, 27.3, 27.1, 26.1, 25.2, 24.9.

HRMS (ESI): calc. for $C_{50}H_{60}N_9O_9S_4^+$ $[M]^+$: 1058.3391, found: 1058.3401.

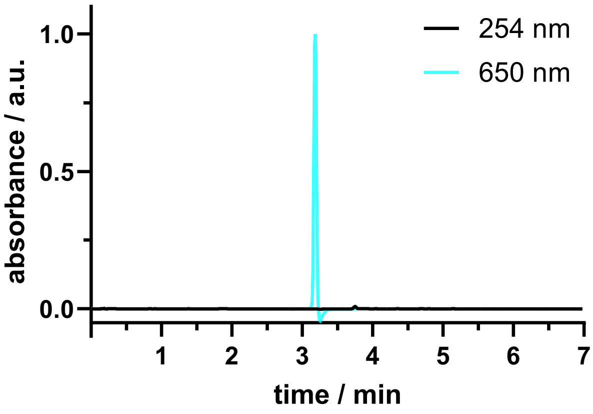

3.5 2-((*E*)-3-((*E*)-1-(27-chloro-6,14-dioxo-18,21-dioxa-10,11-dithia-7,15-diazaheptacosyl)-3,3-dimethyl-5-sulfoindolin-2-ylidene)prop-1-en-1-yl)-1,3,3-trimethyl-3*H*-indol-1-ium-5-sulfonate (CA-SS-SulfoCy3)

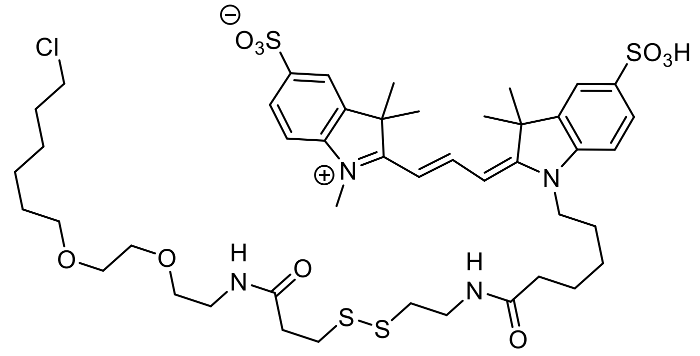

2 (5.4 mg, 6.1 µmol 1.0 equiv.) was dissolved in 1 mL DMSO, before DIPEA (4.1 µL, 24.4 µmol, 4.0 equiv.) and TSTU (2.8 mg, 9.2 µmol, 1.5 equiv.) were added. The reaction was stirred for 30 min at rt, before it was quenched by addition of glacial HOAc (20 µL). The reaction mixture was subjected to RP-HPLC. The product containing fractions were combined, lyophilized, and dissolved in 1 mL DMSO. DIPEA (4.1 µL, 24.4 µmol, 4.0 equiv.) and CA-NH_2 (1.6 mg, 7.32 µmol, 1.2 equiv.) were added. The reaction was stirred for 30 min at rt, before it was quenched by addition of glacial HOAc (20 µL). The reaction mixture was subjected to RP-HPLC to obtain 5.3 mg (4.8 µmol) of the desired compound as a red powder in 79 % yield.

^{1}H NMR (600 MHz, MeOD-d$_4$): δ [ppm] = 8.35 (t, *J* = 13.5 Hz, 1H), 7.98 (m, *J* = 5.2 Hz, 3H), 7.80 (t, *J* = 1.8 Hz, 2H), 7.68 (m, *J* = 2.7 Hz, 2H), 7.38 (t, *J* = 8.6 Hz, 2H), 6.48 (dd, *J* = 5.0, 13.4 Hz, 2H), 4.10 (t, *J* = 7.4 Hz, 2H), 3.66 (s, 3H), 3.61 (t, *J* = 6.6 Hz, 2H), 3.47 (m, *J* = 3.3 Hz, 4H), 3.39 (t, *J* = 5.9 Hz, 2H), 3.36 (t, *J* = 6.6 Hz, 2H), 3.29 (q, *J* = 6.4 Hz, 2H), 3.19 (q, *J* = 5.8 Hz, 2H), 2.87 (t, *J* = 7.2 Hz, 2H), 2.74 (t, *J* = 6.8 Hz, 2H), 2.45 (t, *J* = 7.2 Hz, 2H), 2.07 (t, *J* = 7.3 Hz, 2H), 1.76-1.67 (m, 4H), 1.70 (s, 12H), 1.57 (q, *J* = 7.5 Hz, 2H), 1.47 (q, *J* = 7.0 Hz, 2H), 1.37 (q, *J* = 6.0 Hz, 4H), 1.30 (q, *J* = 3.0 Hz, 2H).

^{13}C NMR (151 MHz, MeOD-d$_4$): δ [ppm] = 175.3, 174.4, 173.4, 172.5, 170.5, 150.1, 146.4, 146.2, 143.0, 142.3, 140.4, 126.7, 126.5, 120.3, 120.2, 111.0, 103.8, 103.2, 70.6, 70.0, 69.8, 69.7, 69.5, 65.9, 63.0, 49.3, 45.8, 44.3, 39.0, 38.3, 37.7, 35.5, 35.4, 34.4, 33.9, 32.4, 32.0, 31.7, 29.5, 29.4, 29.3, 29.1, 28.9, 27.9, 27.7, 27.1, 26.5, 26.2, 25.3, 25.3, 24.9.

HRMS (ESI): calc. for $C_{45}H_{66}ClN_4O_{10}S_4^+$ [M]$^+$: 985.3345, found: 985.3352.

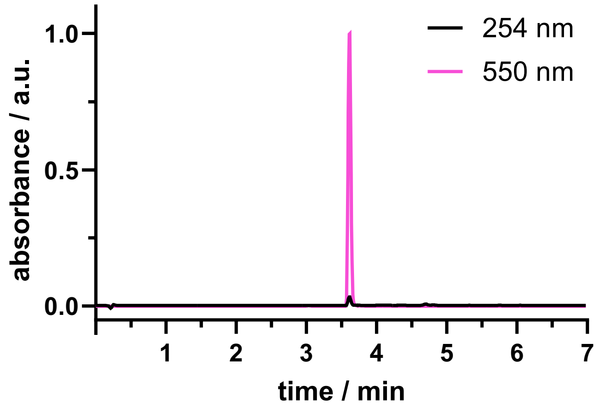

3.6 2-((1E,3E)-5-((E)-1-(27-chloro-6,14-dioxo-18,21-dioxa-10,11-dithia-7,15-diazaheptacosyl)-3,3-dimethyl-5-sulfoindolin-2-ylidene)penta-1,3-dien-1-yl)-1,3,3-trimethyl-3H-indol-1-ium-5-sulfonate (CA-SS-SulfoCy5)

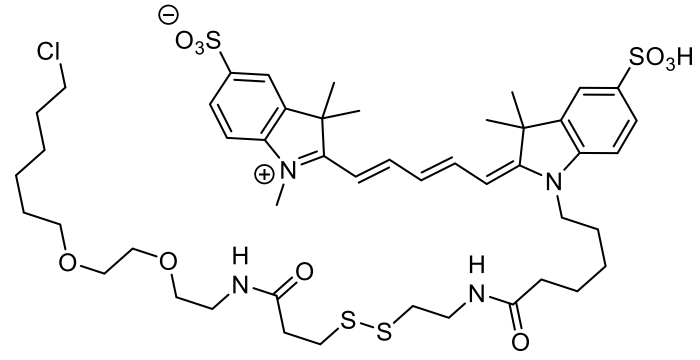

4 (5.0 mg, 5.6 µmol 1.0 equiv.) was dissolved in 1 mL DMSO, before DIPEA (4.0 µL, 23.2 µmol, 4.0 equiv.) and TSTU (2.6 mg, 8.7 µmol, 1.5 equiv.) were added. The reaction was stirred for 30 min at rt, before it was quenched by addition of glacial HOAc (20 µL). The reaction mixture was subjected to RP-HPLC. The product containing fractions were combined, lyophilized, and dissolved in 1 mL DMSO. DIPEA (4.0 µL, 23.2 µmol, 4.0 equiv.) and CA-NH_2 (1.5 mg, 6.7 µmol, 1.2 equiv.) were added and the reaction was stirred for 30 min at rt, before it was quenched by addition of glacial HOAc (20 µL). The reaction mixture was subjected to RP-HPLC to obtain 4.4 mg (3.9 µmol) of the desired compound as a red powder in 70% yield.

^{1}H NMR (600 MHz, MeOD-d_4): δ [ppm] = 8.33 (dt, J = 13.0, 3.6, Hz, 2H), 7.93-7.89 (m, 4H), 7.35 (dd, J = 5.5, 8.2 Hz, 2H), 6.70 (t, J = 12.4 Hz, 1H), 6.35 (d, J = 13.7 Hz, 2H), 4.14 (t, J = 7.3 Hz, 2H), 3.67 (s, 3H), 3.62-3.58 (m, 4H), 3.57 (t, J = 6.5 Hz, 2H), 3.55 (t, J = 5.1 Hz, 2H), 3.49 (t, J = 6.1 Hz, 4H), 3.48 (t, J = 6.0 Hz, 2H), 3.37 (t, J = 5.5 Hz, 2H), 2.97 (t, J = 7.2 Hz, 2H), 2.82 (t, J = 6.6 Hz, 2H), 2.62 (t, J = 7.1 Hz, 2H), 2.24 (t, J = 7.2 Hz, 2H), 1.86 (p, J = 7.6 Hz, 2H), 1.77 (s, 12H), 1.73 (p, J = 7.7 Hz, 2H), 1.60 (m, J = 7.0 Hz, 2H), 1.52-1.45 (m, 4H), 1.41 (p, J = 4.7 Hz, 2H).

^{13}C NMR (151 MHz, MeOD-d_4): δ [ppm] = 174.1, 173.4, 173.3, 172.5, 170.5, 154.6, 145.8, 145.7, 143.1, 142.4, 140.9, 140.8, 126.5, 126.4, 126.1, 120.4, 120.3, 110.5, 110.5, 104.0, 103.7, 70.6, 70.0, 69.8, 69.7, 69.5, 65.9, 63.1, 49.3, 45.8, 43.8, 39.0, 38.3, 37.7, 35.5, 35.4, 34.4, 33.9, 32.4, 31.7, 31.6, 29.5, 29.4, 29.3, 29.1, 28.9, 27.5, 27.3, 27.1, 26.5, 26.1, 25.3, 25.3, 24.9.

HRMS (ESI): calc. for $C_{47}H_{68}ClN_4O_{10}S_4^+$ $[M]^+$: 1011.3501, found: 1011.3522.

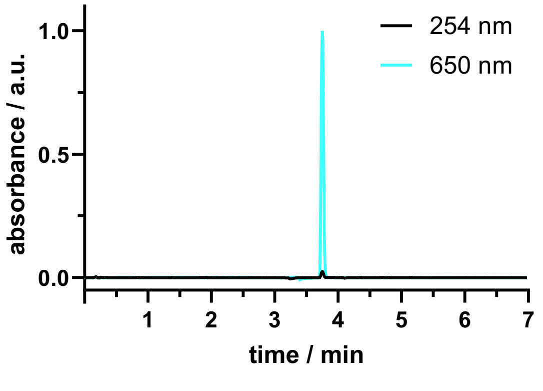

4 NMR-Spectra

4.1 2-((*E*)-3-((*E*)-1-(6-((2-((2-carboxyethyl)disulfaneyl)ethyl)amino)-6-oxohexyl)-3,3-dimethyl-5-sulfoindolin-2-ylidene)prop-1-en-1-yl)-1,3,3-trimethyl-3*H*-indol-1-ium-5-sulfonate (2)

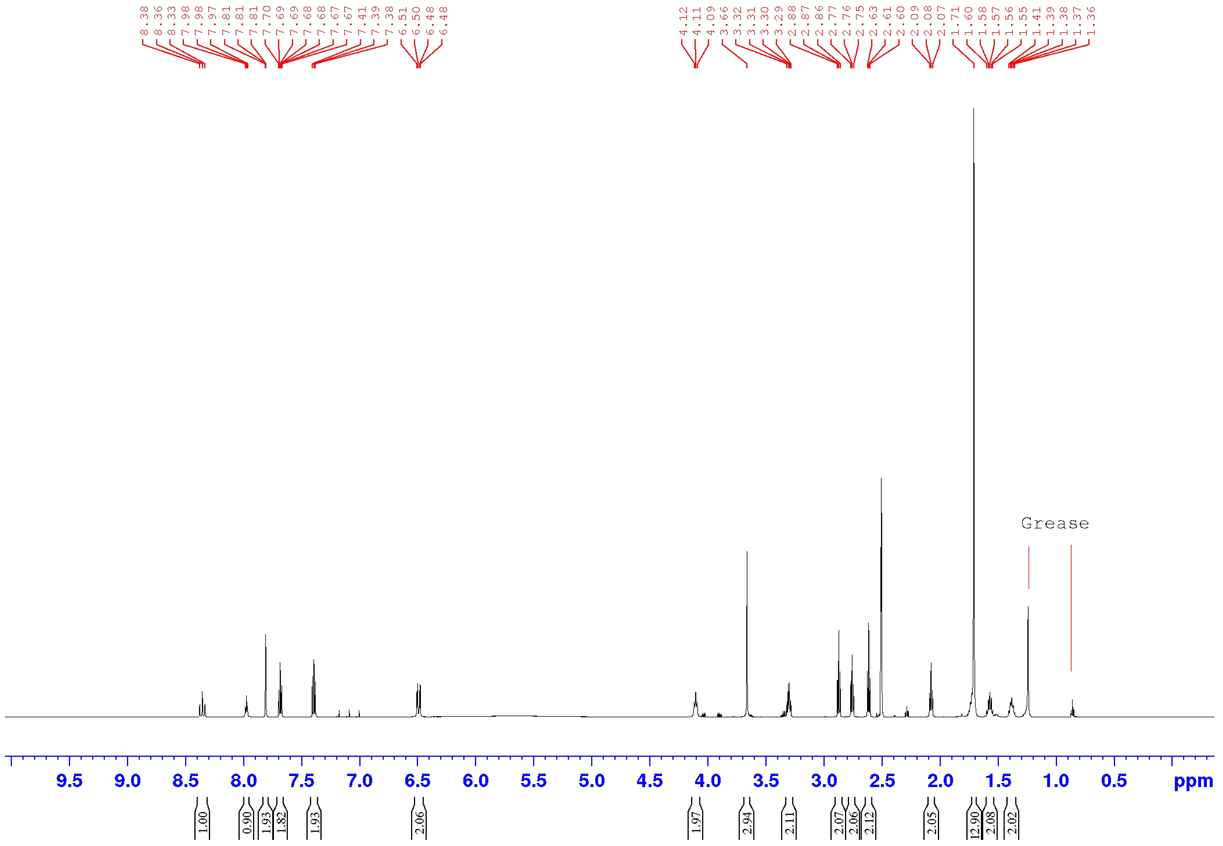

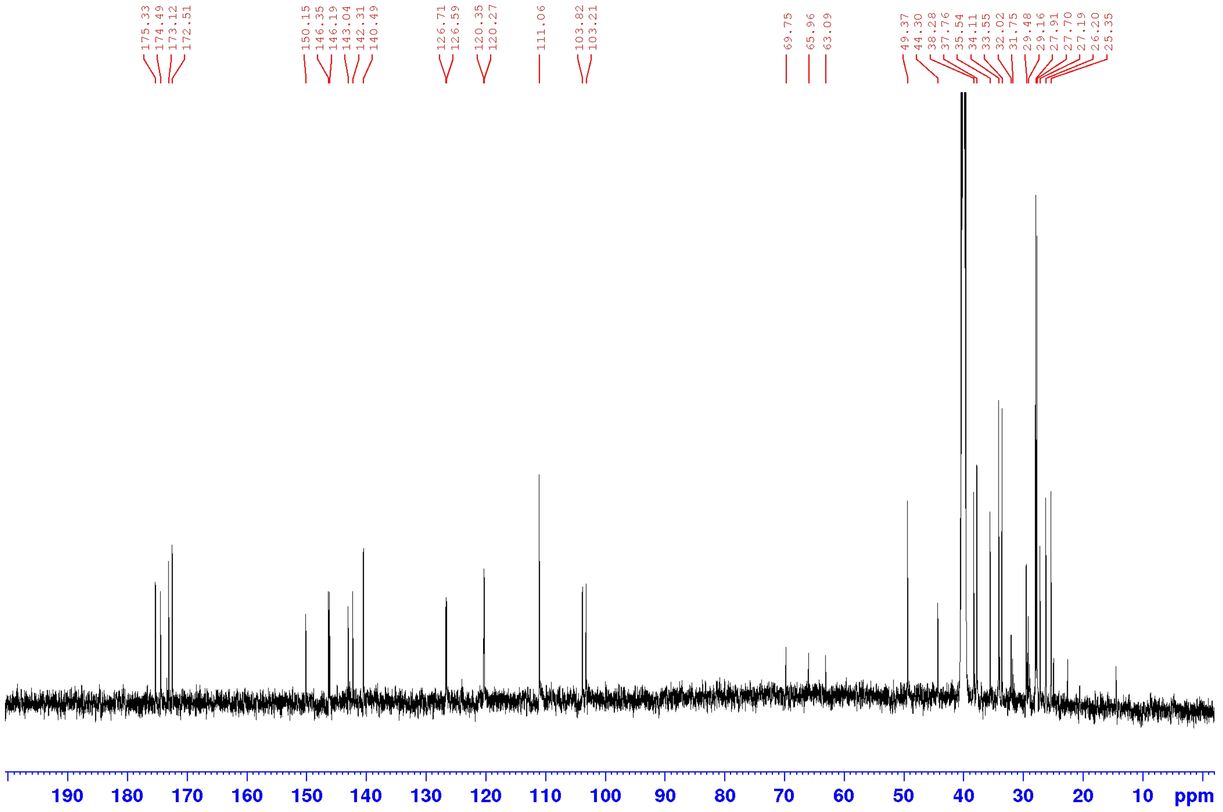

4.2 2-((1*E*,3*E*)-5-((*E*)-1-(6-((2-((2-carboxyethyl)disulfaneyl)ethyl)amino)-6-oxohexyl)-3,3-dimethyl-5-sulfoindolin-2-ylidene)penta-1,3-dien-1-yl)-1,3,3-trimethyl-3*H*-indol-1-ium-5-sulfonate (4)

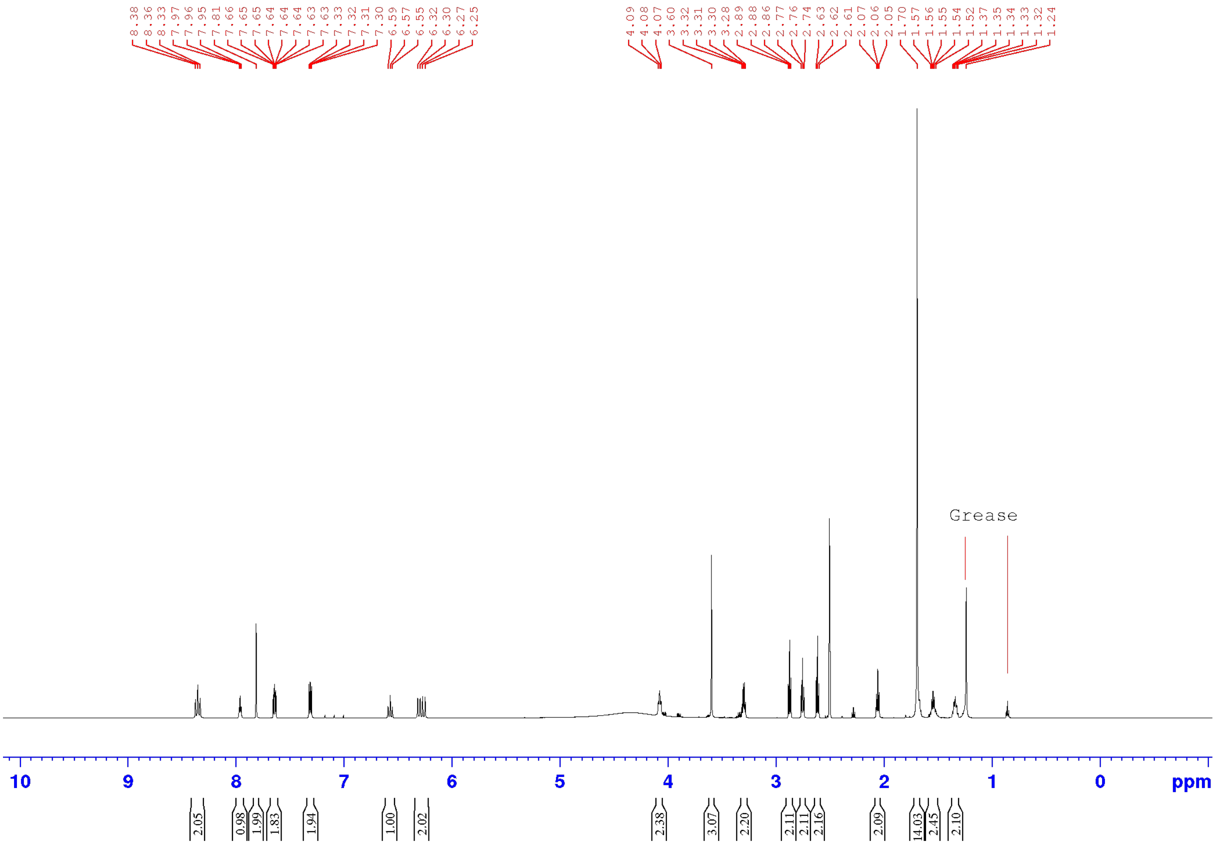

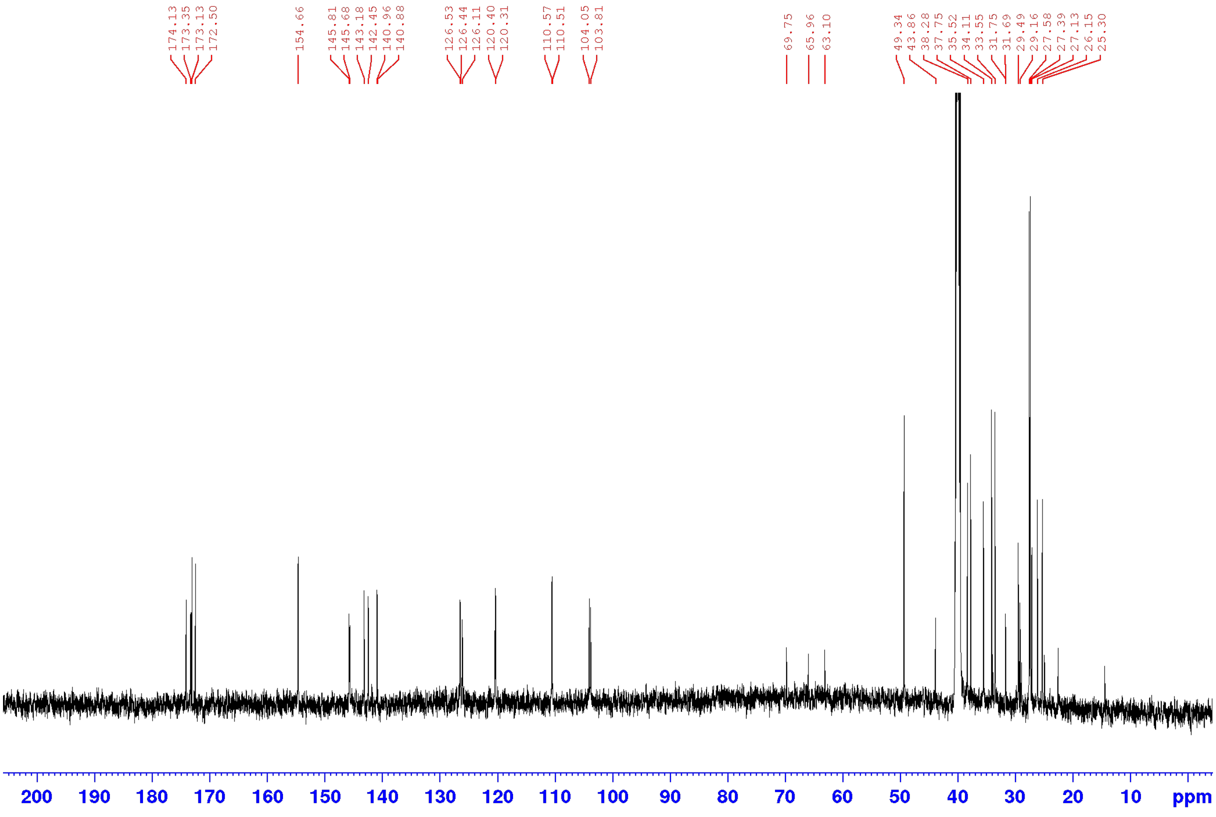

4.3 2-((*E*)-3-((*E*)-1-(6-((2-((3-((4-(((2-amino-9*H*-purin-6-yl)oxy)methyl)benzyl)amino)-3-oxopropyl)disulfaneyl)ethyl)amino)-6-oxohexyl)-3,3-dimethyl-5-sulfoindolin-2-ylidene)prop-1-en-1-yl)-1,3,3-trimethyl-3*H*-indol-1-ium-5-sulfonate (BG-SS-SulfoCy3)

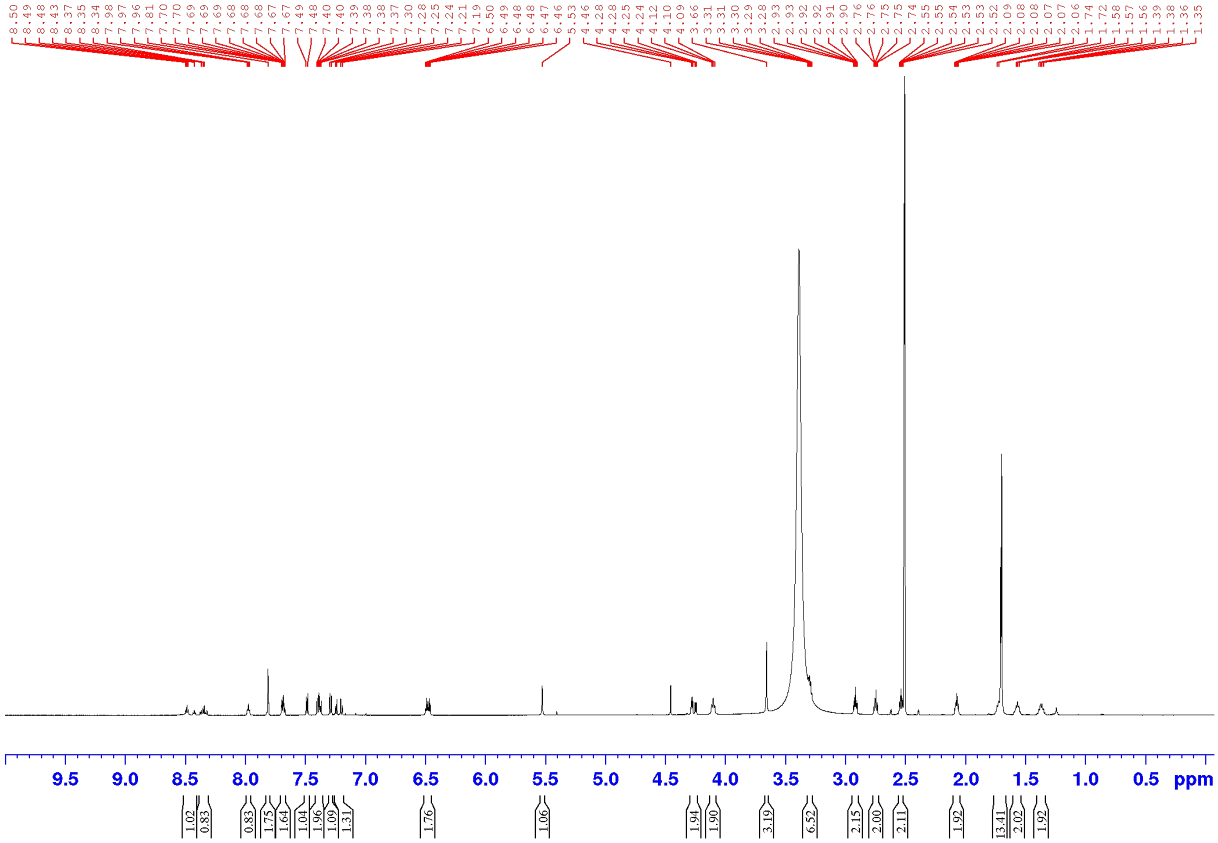

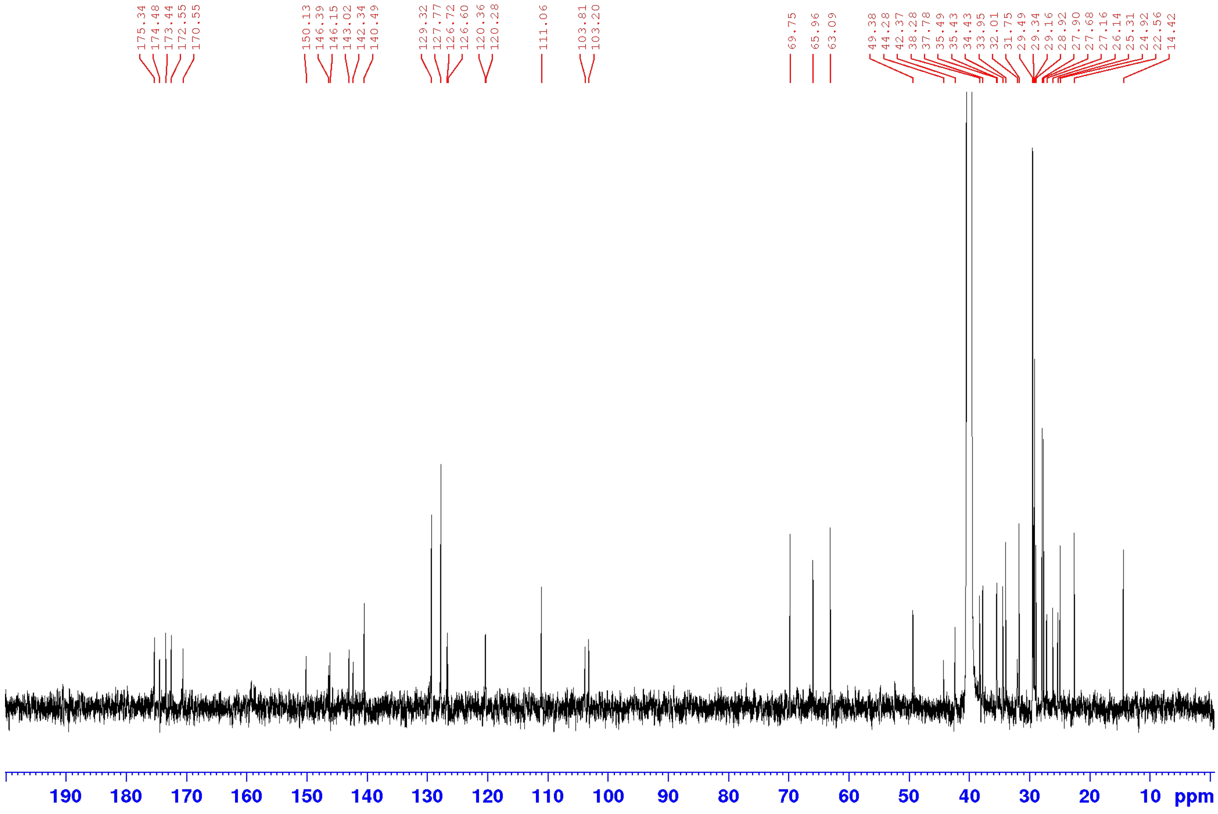

4.4 2-((1*E*,3*E*)-5-((*E*)-1-(6-((2-((3-((4-(((2-amino-9*H*-purin-6-yl)oxy)methyl)benzyl)amino)-3-oxopropyl)disulfaneyl)ethyl)amino)-6-oxohexyl)-3,3-dimethyl-5-sulfoindolin-2-ylidene)penta-1,3-dien-1-yl)-1,3,3-trimethyl-3*H*-indol-1-ium-5-sulfonate (BG-SS-SulfoCy5)

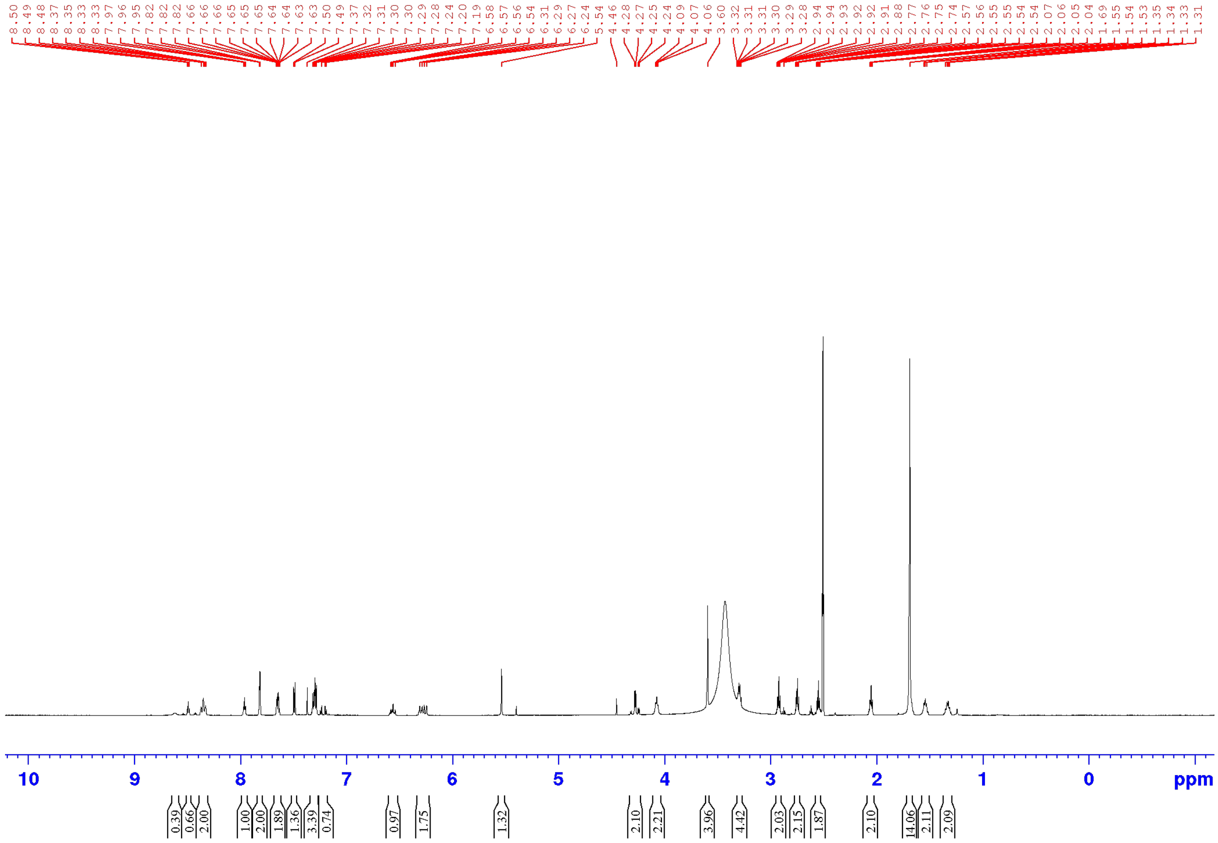

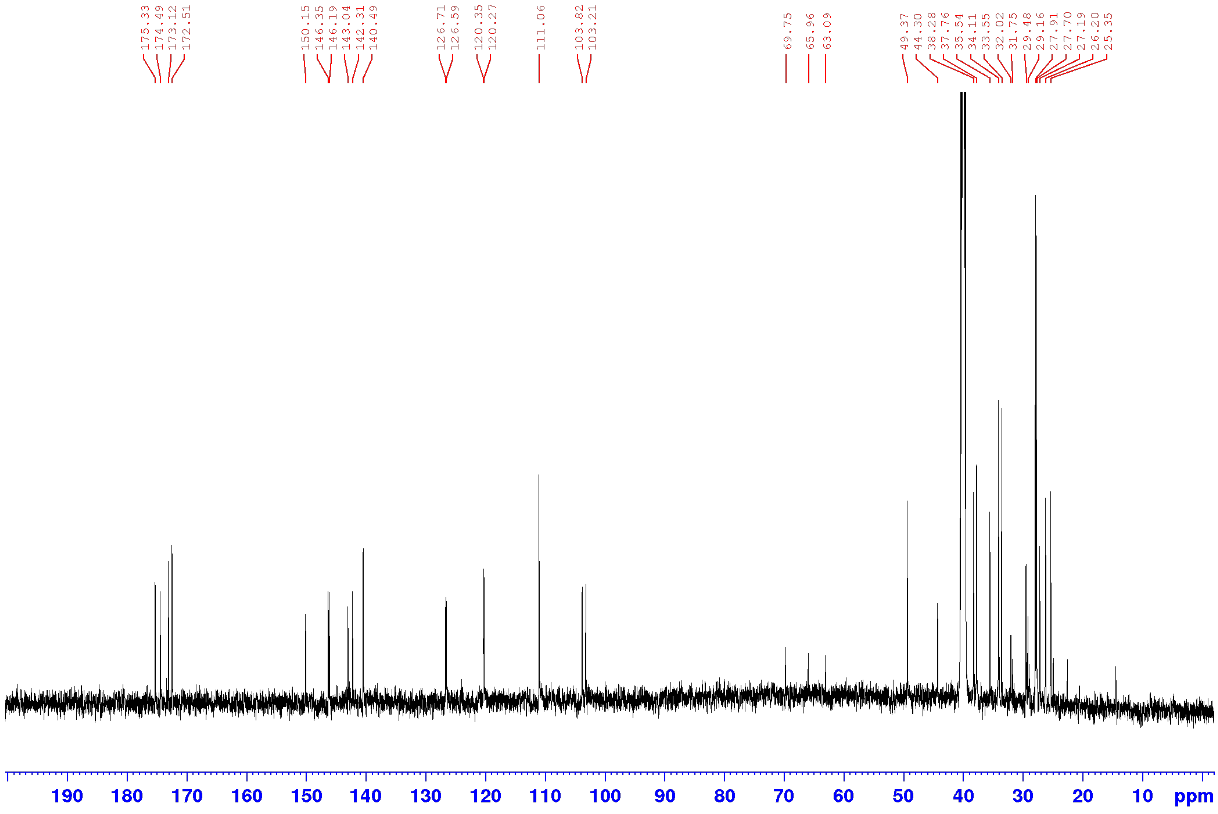

4.5 2-((*E*)-3-((*E*)-1-(27-chloro-6,14-dioxo-18,21-dioxa-10,11-dithia-7,15-diazaheptacosyl)-3,3-dimethyl-5-sulfoindolin-2-ylidene)prop-1-en-1-yl)-1,3,3-trimethyl-3*H*-indol-1-ium-5-sulfonate (CA-SS-SulfoCy3)

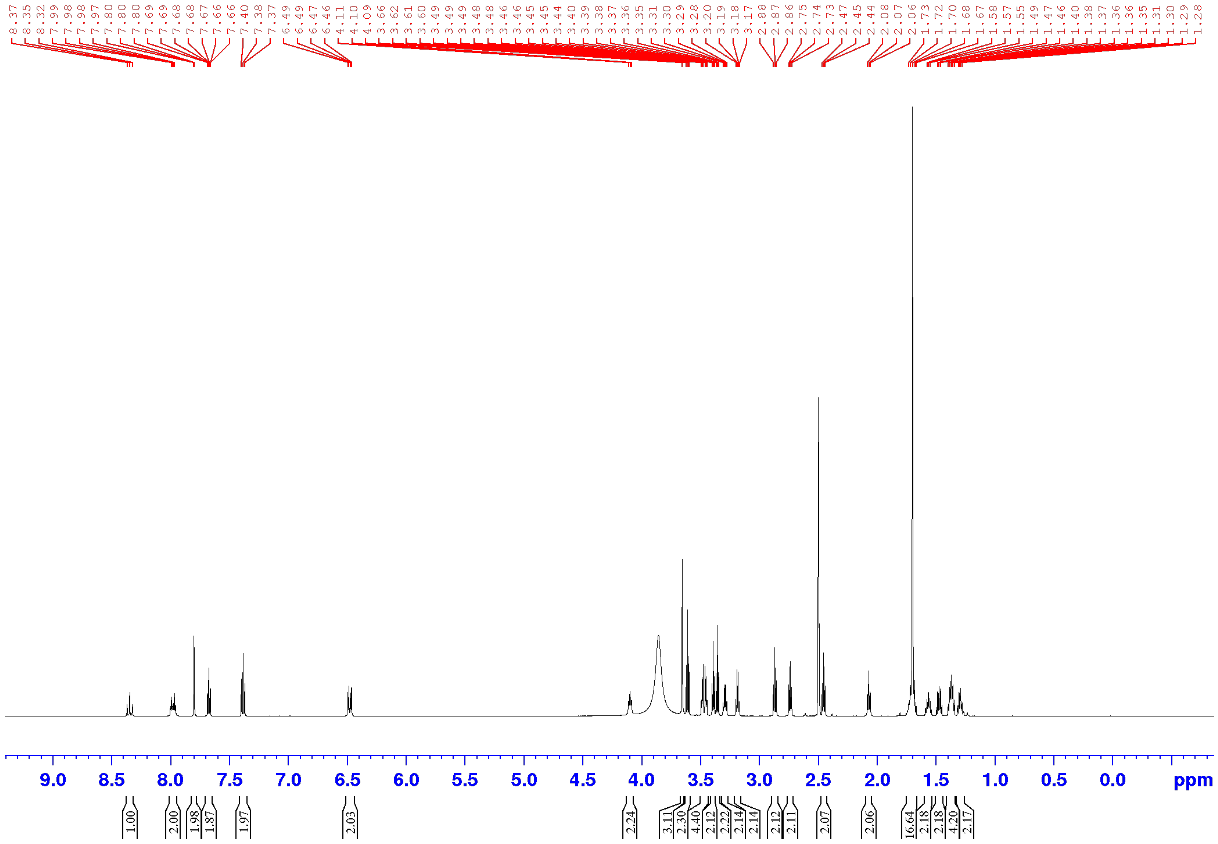

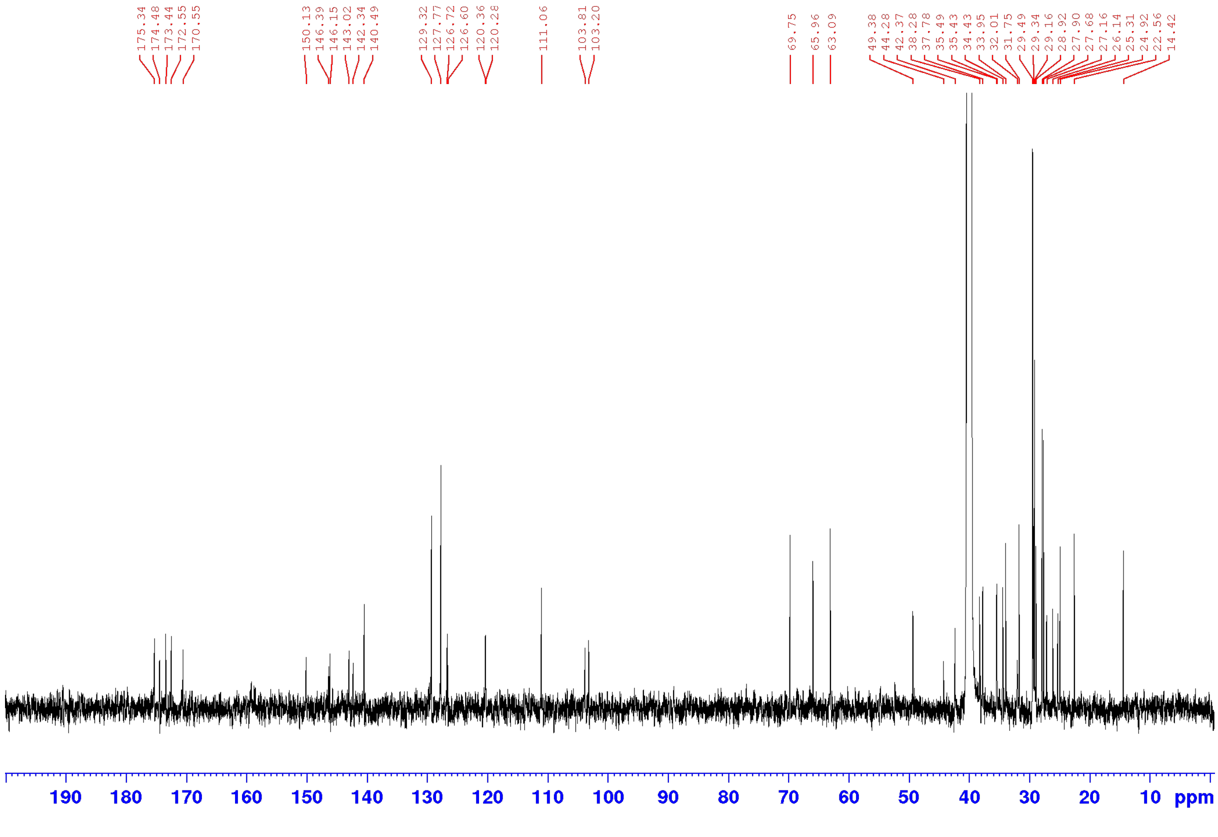

4.6 2-((1*E*,3*E*)-5-((*E*)-1-(6-((2-((3-((4-(((2-amino-9*H*-purin-6-yl)oxy)methyl)benzyl)amino)-3-oxopropyl)disulfaneyl)ethyl)amino)-6-oxohexyl)-3,3-dimethyl-5-sulfoindolin-2-ylidene)penta-1,3-dien-1-yl)-1,3,3-trimethyl-3*H*-indol-1-ium-5-sulfonate (CA-SS-SulfoCy5)

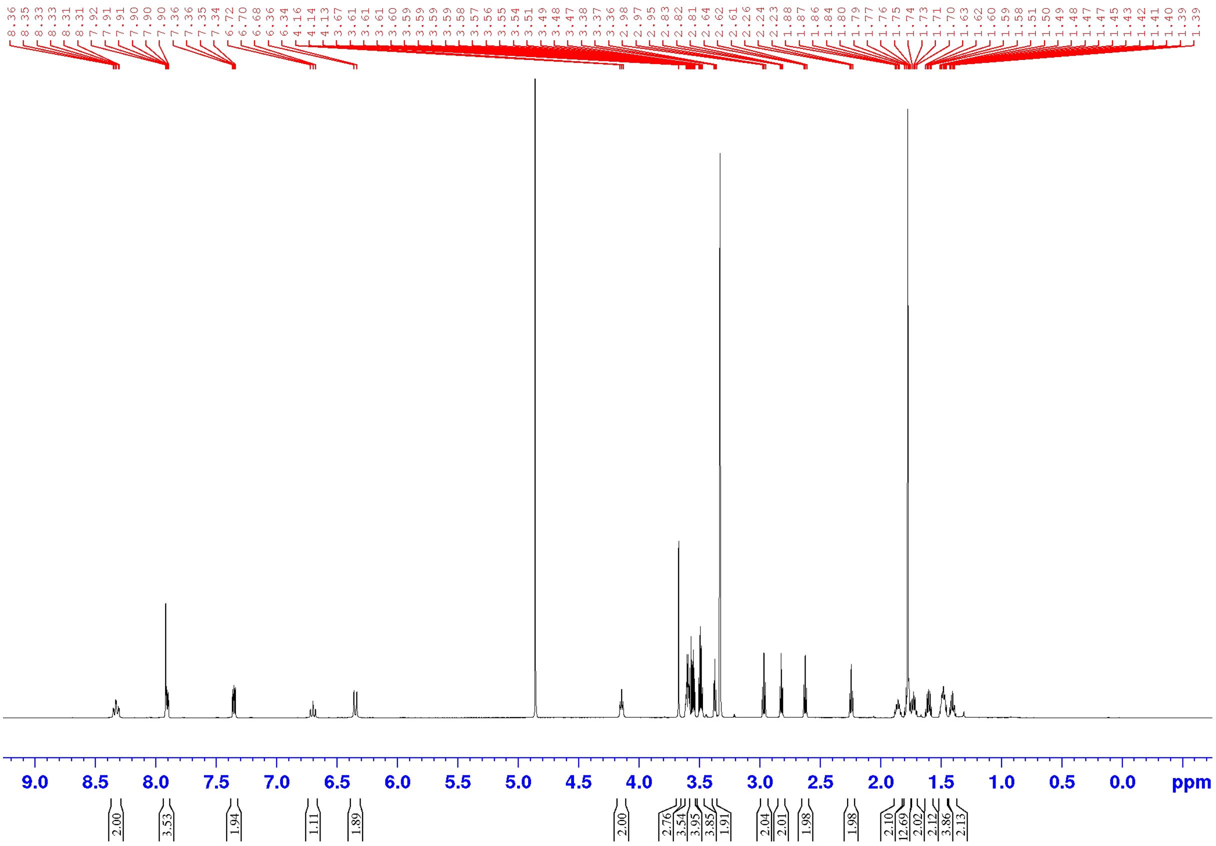

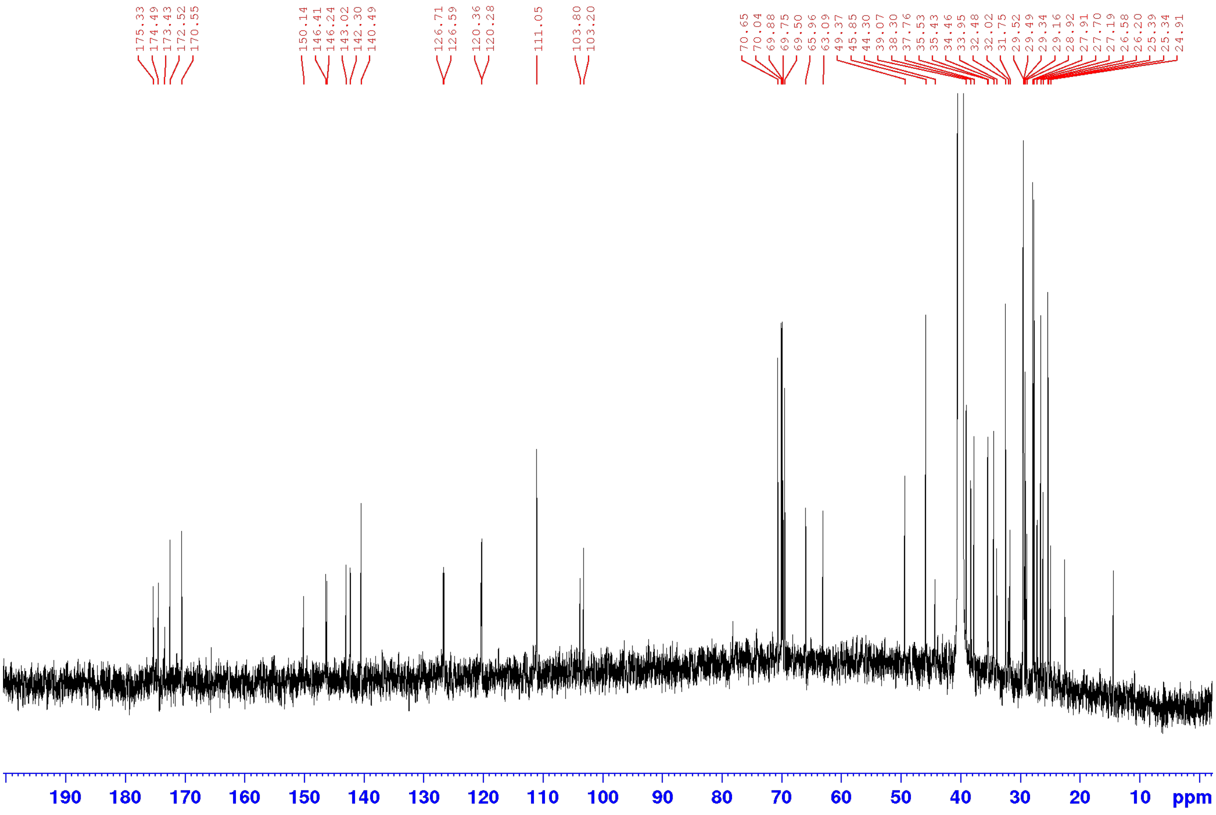

5 References

(1) Fang, Z.; Chen, S.; Manchanda, Y.; Bitsi, S.; Pickford, P.; David, A.; Shchepinova, M. M.; Corrêa, I. R., Jr.; Hodson, D. J.; Broichhagen, J.; Tate, E. W.; Reimann, F.; Salem, V.; Rutter, G. A.; Tan, T.; Bloom, S. R.; Tomas, A.; Jones, B. Ligand-Specific Factors Influencing GLP-1 Receptor Post-Endocytic Trafficking and Degradation in Pancreatic Beta Cells. *Int J Mol Sci* **2020,** *21* (21).

(2) Peng, T.; Thorn, K.; Schroeder, T.; Wang, L.; Theis, F. J.; Marr, C.; Navab, N. A BaSiC tool for background and shading correction of optical microscopy images. *Nature Communications* **2017,** *8* (1), 14836.

(3) Jaccard, N.; Griffin, L. D.; Keser, A.; Macown, R. J.; Super, A.; Veraitch, F. S.; Szita, N. Automated method for the rapid and precise estimation of adherent cell culture characteristics from phase contrast microscopy images. *Biotechnol Bioeng* **2014,** *111* (3), 504.
